# Development of a blood-based molecular biomarker test for identification of schizophrenia before disease onset

**DOI:** 10.1038/tp.2015.91

**Published:** 2015-07-14

**Authors:** M K Chan, M-O Krebs, D Cox, P C Guest, R H Yolken, H Rahmoune, M Rothermundt, J Steiner, F M Leweke, N J M van Beveren, D W Niebuhr, N S Weber, D N Cowan, P Suarez-Pinilla, B Crespo-Facorro, C Mam-Lam-Fook, J Bourgin, R J Wenstrup, R R Kaldate, J D Cooper, S Bahn

**Affiliations:** 1 Department of Chemical Engineering and Biotechnology, University of Cambridge, Cambridge, UK; 2 INSERM UMR 894, Centre of Psychiatry and Neurosciences, Lab Pathophysiology of Psychiatric Disorders, Institut de Psychiatrie (GDR 3557) Paris, France; 3 University Paris Descartes, Sorbonne Paris Cité, Faculty of Medicine Paris Descartes, Service Hospitalo-Universitaire, Centre hospitalier Sainte-Anne, Paris, France; 4 Johns Hopkins University School of Medicine, Baltimore, MD, USA; 5 University of Muenster, Germany and Evangelisches Klinikum Niederrhein, Oberhausen, Germany; 6 Department of Psychiatry, University of Magdeburg, Magdeburg, Germany; 7 Central Institute of Mental Health, Medical Faculty Mannheim, University of Heidelberg, Mannheim, Germany; 8 Department of Neuroscience, Erasmus MC, Rotterdam, The Netherlands; 9 Walter Reed Army Institute of Research, Silver Spring, MD, USA; 10 CIBERSAM, University Hospital Marqués de Valdecilla, Department of Psychiatry, University of Cantabria - IDIVAL, Santander, Spain; 11 Myriad Genetic Laboratories, Inc., Salt Lake City, UT, USA

## Abstract

Recent research efforts have progressively shifted towards preventative psychiatry and prognostic identification of individuals before disease onset. We describe the development of a serum biomarker test for the identification of individuals at risk of developing schizophrenia based on multiplex immunoassay profiling analysis of 957 serum samples. First, we conducted a meta-analysis of five independent cohorts of 127 first-onset drug-naive schizophrenia patients and 204 controls. Using least absolute shrinkage and selection operator regression, we identified an optimal panel of 26 biomarkers that best discriminated patients and controls. Next, we successfully validated this biomarker panel using two independent validation cohorts of 93 patients and 88 controls, which yielded an area under the curve (AUC) of 0.97 (0.95–1.00) for schizophrenia detection. Finally, we tested its predictive performance for identifying patients before onset of psychosis using two cohorts of 445 pre-onset or at-risk individuals. The predictive performance achieved by the panel was excellent for identifying USA military personnel (AUC: 0.90 (0.86–0.95)) and help-seeking prodromal individuals (AUC: 0.82 (0.71–0.93)) who developed schizophrenia up to 2 years after baseline sampling. The performance increased further using the latter cohort following the incorporation of CAARMS (Comprehensive Assessment of At-Risk Mental State) positive subscale symptom scores into the model (AUC: 0.90 (0.82–0.98)). The current findings may represent the first successful step towards a test that could address the clinical need for early intervention in psychiatry. Further developments of a combined molecular/symptom-based test will aid clinicians in the identification of vulnerable patients early in the disease process, allowing more effective therapeutic intervention before overt disease onset.

## Introduction

Diagnosis of schizophrenia has not changed over the last 100 years since Emil Kraepelin first defined the disease and is still based on evaluation of signs and symptoms in clinical interviews. If a patient does not acknowledge the occurrence of symptoms of psychosis, such as hallucinations and delusions, the disease can remain undiagnosed. In addition, some of the symptoms can also occur in patients with mood and personality disorders and, therefore, misdiagnosis is a common occurrence. For example, Gonzalez-Pinto *et al.*^1^ found that approximately one-third of bipolar patients were diagnosed with schizophrenia or other psychotic disorders, particularly in youths with short medical histories. Another complication and reason for the delay in diagnosis of schizophrenia is the insidious disease onset and the possibility of multiple or combinatorial causes in the development or manifestation of the disease.

Over the last two decades, prodromal schizophrenia has become a major focus of psychiatric research. This condition is also known as ultra-high-risk syndrome and is normally characterized using structured clinical interviews between patients and psychiatrists for the evaluation of disturbances in perception, thought processing, language and attention.^2^ Investigations have shown that 20–30% of these individuals eventually develop schizophrenia over a 2–3-year period.^3^ Early diagnosis of schizophrenia would be beneficial for the outcome of patients, especially if this could be achieved before or during the prodromal stages. This is due to the fact that shorter periods of untreated psychosis have been linked to better patient outcomes.^4^ In line with this, the recent revision of the Diagnostic and Statistical Manual of Mental Disorders (DSM-5) has led to discussions on the prodromal syndrome as a potential diagnostic category and this has now been listed in the DSM-5 appendix as a ‘condition for further systematic study'.^5^ However, there is still concern that an incorrect diagnosis could result in unwarranted treatment and stigma as ~70% of individuals who fulfil prodromal criteria do not develop schizophrenia.^2^ These concerns highlight the pressing need to identify robust biomarkers for detection of schizophrenia before disease onset.

As a first step towards addressing this problem, Schwarz *et al.*^6^ reported on the identification of inflammatory, oxidative stress and hypothalamic–pituitary–adrenal signalling serum proteins altered in first-onset schizophrenia patients. The next stages in this research area are to refine and validate such an approach by developing a serum biomarker panel for improved diagnosis and, most importantly, to evaluate whether this could be used to predict the risk of conversion or transition to schizophrenia in at-risk individuals. Recently, Perkins *et al.*^7^ published an algorithm comprising a panel of 15 analytes identified in plasma for prediction of progression of high-risk individuals to psychosis with an AUC of 0.88. However, due to sample-size limitations, this algorithm was trained and tested on the same sample set, examining individuals who did (*n*=32) or did not (*n*=40) progress to psychosis and controls (*n*=35).

We believe this is the first study using a multistage approach to identify a serum biomarker panel in serum of first-onset patients for the identification of individuals at risk of developing schizophrenia. The first stage involved meta-analysis of five independent cohorts comprising 331 first-onset drug-naive schizophrenia patients and controls to establish a diagnostic serum biomarker panel. The next stage involved validation of this panel using two additional independent cohorts of 181 schizophrenia patients and controls. The third stage was the unbiased application of this panel to predict development of schizophrenia in two further independent cohorts of 445 pre-onset or help-seeking prodromal individuals who were sampled months to years before disease onset and diagnosis.

## Materials and methods

### Clinical cohorts

For the first phase (discovery phase) of the study, individuals were recruited consecutively from two clinical centres in Germany (cohort 1, Central Institute of Mental Health, Mannheim; cohorts 2–4, University of Magdeburg, Magdeburg) and one in the Netherlands (cohort 5, Erasmus University MC, Rotterdam). All the patients in cohorts 1–5 were diagnosed as having the paranoid subtype of schizophrenia (295.30). The samples were a subset of those used in Schwarz *et al.*^6^ and were selected only to include first- or recent-onset antipsychotic-naive schizophrenia patients and controls with the best matching of demographic characteristics as indicated in Table 1. For the second phase (validation phase), individuals were recruited consecutively from clinics in Germany (cohort 6, University of Muenster, Muenster) and Spain (cohort 7, University of Cantabria, Santander; for detailed recruitment information, see Pelayo-Teran *et al.*^8^; Table 1). Patients from these cohorts were first- or recent-onset and antipsychotic-naive or unmedicated at the time of sample collection. For both phases, DSM-IV diagnosis was performed by psychiatrists and additional analysis included Positive and Negative Syndrome Scale testing.^9^ The inter-rater variability was <10% across the sites and recruitment periods spanned for up to a decade. Information on antipsychotic medication use was confirmed by direct contact with the treating family physicians, relatives and spouses along with consultations regarding detailed current histories of psychotropic medication use before hospitalization. Controls were recruited simultaneously from the community through advertisements or selected from a clinical database of volunteers (students, staff, relatives of staff and blood donors from local blood banks) and matched with the respective patient groups for age, gender and other patient characteristics such as body mass index, smoking and cannabis, when this information was available (Table 1). For both patients and controls, the exclusion criteria included: those having first-degree relatives with a medical history of mental disease, diabetes, cardiovascular disease, immune and autoimmune disorders, infections, treatment with immunosuppressive/-modulating drugs or antibiotics, other neuropsychiatric/neurological disorders (multiple sclerosis, epilepsy, mental retardation), chronic (terminal) diseases affecting the brain (cancer, hepatic and renal insufficiency), alcohol or drug addiction, organic psychosis/organic affective syndromes, severe trauma, other psychiatric and non-psychiatric co-morbidity. Exclusions were based on examination of current medical histories, rating scales, physical examination, blood tests, magnetic resonance imaging or computed tomography scans, where possible. Medication was administered after completion of diagnostic evaluation as appropriate. In addition, informed written consent was given by all participants and the study protocols, analysis of samples and test methods were approved by the local Institutional Ethics Review Boards and were in compliance with the Standards for Reporting of Diagnostic Accuracy.^10^

For the third phase of the study, retrospective samples were used, which were obtained from individuals who were later diagnosed with schizophrenia or bipolar disorder. One set of samples (cohort 8) was selected from the US Department of Defense Serum Repository (DoDSR), which contains over 55 million serum specimens remaining from mandatory HIV test samples of military personnel. Data and sera retrieval for two larger nested case–control studies were performed by the Armed Forces Health Surveillance Center (AFHSC) and coordinated by the Military New-Onset Psychosis Project (MNOPP) investigators at the Walter Reed Army Institute of Research. The medical and demographic data were provided by the Defense Medical Surveillance System, AFHSC, US DoD, Silver Spring, Maryland (the data ranged from 1971 to 2006 and was released in 2007) and serum samples were retrieved from the DoDSR, AFHSC, US DoD (Silver Spring, MD, USA; the samples ranged from 1988 to 2006 and were released in 2007). Sera were then transferred to the Johns Hopkins School of Medicine (Baltimore, MD, USA) before testing. At the time of sample collection, the military personnel had not presented with psychiatric symptoms. Samples were then selected from 185 individuals who later presented with psychiatric symptoms within 30 days after blood collection and then received a DSM-IV diagnosis of either schizophrenia (pre-schizophrenia; 295.10–295.30, 295.60, 295.70, 295.90) or bipolar disorder (pre-bipolar disorder; 296.00–296.06, 296.40–296.7, 296.80, 296.89; MNOPP; Table 1).^12, 13^ The diagnostic process leading to medical discharge from military service and validity of the psychiatric diagnosis has been detailed elsewhere.^13^ Control individuals were selected from active duty military service population with no inpatient or outpatient psychiatric disorder diagnoses, as confirmed by current military records. All data were previously collected for other purposes, and analyses were conducted on de-identified data. An informed consent waiver was granted by the Institutional Review Board as only de-identified data were utilized in the study.

Cohort 9 consisted of 76 individuals who were referred consecutively to the Adolescent and Young Adults Assessment Center (SHU, Paris, France) between 2009 and 2013 and enrolled in the ICAAR collaborative study. Inclusion criteria included altered global functioning (Social and Occupational Functioning Assessment Scale (SOFAS) <70) associated with psychiatric symptoms and/or subjective cognitive complaints, during the last year. Individuals were excluded who met the DSM-IV-defined criteria for psychosis, schizophrenia or schizo-affective disorders, pervasive developmental or bipolar disorders, as were individuals with other established diagnoses such as obsessive-compulsive disorders. Other exclusion criteria were current antipsychotic treatment for more than 12 weeks, psychoactive substance dependence or abuse during the previous year and/or more than 5 years, serious or evolutive somatic and neurological disorders, head injury and intelligence quotient <70, and non-French-native speaking status. The Comprehensive Assessment of At-Risk Mental State (CAARMS) was conducted by specifically trained psychiatrists.^14^ Among the 76 help-seeking prodromal individuals, 50 met the CAARMS threshold criteria for ultra-high risk and 26 did not (Supplementary Information 1).^15^ Of the 50 individuals who met the CAARMS criteria, 14 later developed schizophrenia and 36 did not. Of the 26 individuals who did not meet the CAARMS criteria, 4 developed schizophrenia and 22 did not. This resulted in a total of 18 help-seeking prodromal/non-prodromal individuals who later developed schizophrenia and 58 who did not. Clinical symptoms were assessed using the Brief Psychiatric Rating Scale 24-item extended version with anchor.^16^ As carried out for cohorts 1–7, informed written consent was given by all the participants, and study protocols, collection and analysis of samples and all test methods were approved by the local Institutional Ethics Review Boards.

### Serum sample preparation

Standard operating protocols were prepared for serum sample preparation and used by all the clinical centres, as described previously^6^ (for details, see Supplementary Information 2). Samples were randomized and processed blind to disease status.

### Multiplexed immunoassay analyses

The multi-analyte profiling immunoassay platform was used to measure the concentrations of up to 225 analytes in serum samples from the respective clinical centres. These analytes are involved in various hormonal, immune and inflammatory, metabolic and neurotrophic pathways. All assays were conducted in the Clinical Laboratory Improved Amendments (CLIA)–certified laboratory at Myriad-RBM (Austin, TX, USA), as described previously^6^ (Supplementary Information 3). Instrument performance and assay reproducibility were assessed using quality control samples which had a coefficient of variation <15%. The study protocols, analysis of samples and test methods were carried out in compliance with the Standards for Reporting of Diagnostic Accuracy initiative.^10^

### Statistical analysis and experimental design

All the statistical analyses were performed in R (http://www.R-project.org/).^17^ Multiplex immunoassay data from all the nine cohorts were quality control (QC) assessed and pre-processed to remove analytes with >30% missing values (QC criteria). Missing values are defined as analytes with measurement values below or above the detection limits (Supplementary Table 1). Sample outliers were identified using principal component analysis^18^ through inspection of quantile–quantile plots. Data were imputed as described previously^6^ and log_10_-transformed to stabilize variance.

**Figure 1:**
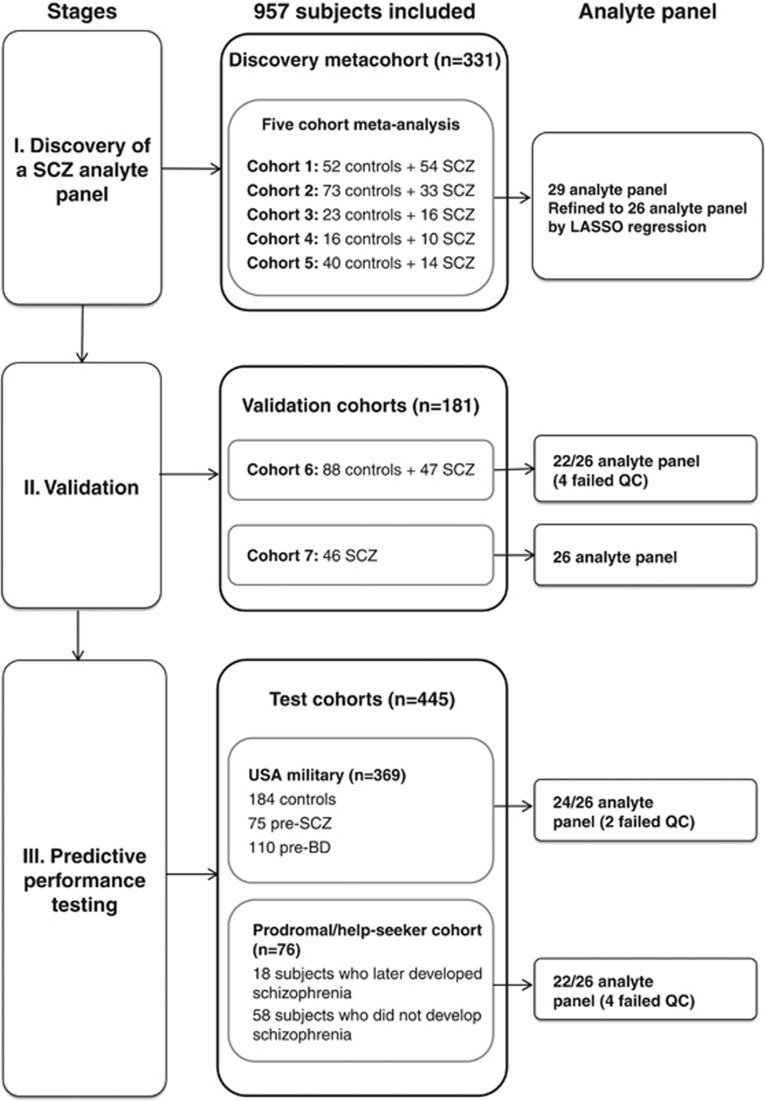
Workflow showing participant inclusion and biomarker panel selection/testing over the three phases of analysis. In stage I, meta-analysis of serum analyte data from cohorts 1–5 was carried out to identify a panel of diagnostic serum biomarkers that discriminates patients from controls using logistic regression. This led to initial identification of 29 significant analytes, which was refined to an optimal set of 26 analytes using the LASSO regression with 10-fold cross-validation. In stage II, the optimal panel was validated using independent validation cohorts. In stage III, predictive performance of the panel was tested in schizophrenia patients before disease onset. Analytes fail QC criteria if they contain over 30% missing values. BD, bipolar disorder; LASSO, least absolute shrinkage and selection operator; QC, quality control; SCZ, schizophrenia.

The overall strategy was divided into three stages (details of participant inclusion and assay selection for the final biomarker panel are summarized in Figure 1). The first stage involved the development of a biomarker panel, comprising the best analytes for discriminating first-onset drug-naive patients from controls. This involved meta-analysis (fixed effects modelling) of cohorts 1–5 resulting in the exclusion of 53 analytes that failed QC in one or more of the cohorts and 27 analytes that were significantly affected by disease-association heterogeneity (Supplementary Tables 2 and 3). Batch effects due to runtime of cohorts were eliminated using the ComBat function in the R package sva.^19^ No sample outliers were identified. The remaining 62 analytes were tested for association with patient/control status (outcome) using logistic regression (age and sex were not significantly associated). Model assumptions for the associated analytes were also tested. False discovery rate was controlled according to Benjamini and Hochberg.^20^ To reduce the model space and examine the joint effects of the analytes, which were significantly associated with patient/control status, we applied least absolute shrinkage and selection operator (LASSO) regression (Supplementary Information 4) with 10-fold cross-validation to select for the optimal set of discriminatory analytes, as implemented in the R package glmnet.^21, 22^ The LASSO approach reduces the coefficients of analytes that have no discriminatory power to zero, while selecting for variables with nonzero coefficients. These variables represent analytes that have high joint discriminatory power to separate patients and controls.^23^

The next stages involved validating the performance of the panel using two independent cohorts of patients and controls (cohorts 6 and 7), and then finally testing the performance for the prediction of schizophrenia development using two separate cohorts of pre-onset and at-risk individuals (cohorts 8 and 9). Predictive performance was evaluated using the test accuracy, sensitivity, specificity, predictive values and the area under the receiver operating characteristic (ROC) curves (AUC) (AUC: 0.9–1.0=excellent; 0.8–0.9=good; 0.7–0.8=fair; 0.6–0.7=poor; 0.5–0.6=fail), using the R package ROCR.^24^ Optimal trade-offs between sensitivity and specificity were determined by maximizing the Youden's index (*J*; calculated by *J*=sensitivity+specificity−1).^25^ One control sample outlier was identified and excluded from cohort 8, through inspection of quantile–quantile plots. Data QC and pre-processing for cohorts 6, 8 and 9 are detailed in Supplementary Tables 4 and 5.

## Results

The study included a total of 957 participants, comprising 331 in the discovery metacohort, 181 in the two validation cohorts and 445 in the pre-onset predictive performance testing cohorts (Table 1, Figure 1). The comparative groups within each cohort were matched for age and sex, and those in the USA military and help-seeker/prodromal cohorts were ~10 years younger compared with those in the discovery and validation cohorts, as these individuals were pre-onset at the time of sampling and therefore likely to be younger than first-onset patients.

### Stage I. Discovery of a first-onset schizophrenia biomarker panel

**Figure 2:**
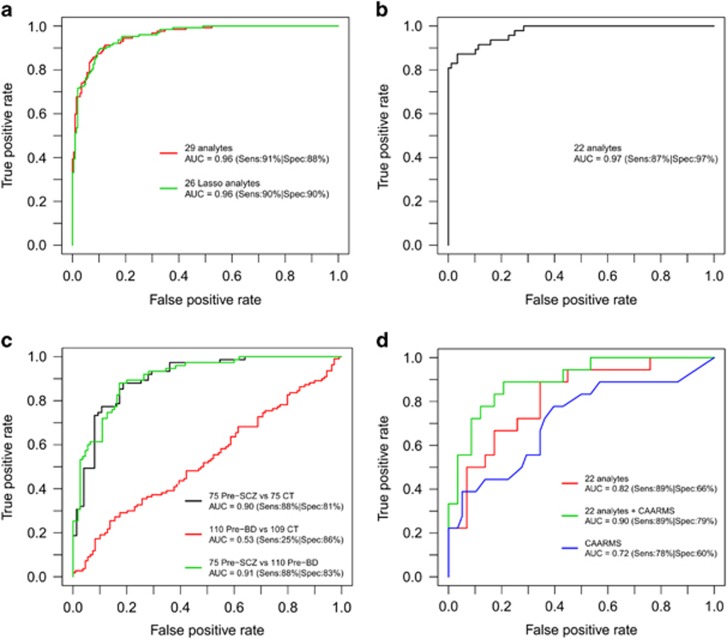
(**a**) ROC curves showing the diagnostic performance achieved using the 29 original analyte combination and the 26 final LASSO-selected SCZ analyte panel in discriminating SCZ patients (*n*=127) from controls (*n*=204) (discovery metacohort). (**b**) ROC curve analysis showing the diagnostic performance achieved using the SCZ analyte panel for discriminating SCZ patients (*n*=47) from controls (*n*=88) from validation cohort 6. (**c**) ROC curve analysis showing diagnostic performance of the SCZ analyte panel in discriminating pre-SCZ military individuals (*n*=75) from controls who did not develop any subsequent psychiatric illness (*n*=75; cohort 8). We then applied the fitted biomarker model on serum data from pre-BD individuals and controls (110 pre-BD, 109 CT) to examine its predictive performance to identify BD before disease onset. This biomarker panel was then further tested for its differential diagnostic performance to discriminate pre-SCZ from pre-BD patients before onset of both diseases. (**d**) ROC curve analysis showing diagnostic performance of the analyte panel for discrimination of help-seeking prodromal individuals who later developed schizophrenia (*n*=18) from those who did not (*n*=58; cohort 9). Note that instead of the full optimal 26-analyte panel, only 22- and 24-analyte panels were tested in figures **b** and **d**, and **c**, respectively. This is due to some analytes failing QC, as described in the methods. AUC, area under curve; BD, bipolar disorder; CAARMS, Comprehensive Assessment of At-Risk Mental State; CT, control; LASSO, least absolute shrinkage and selection operator; QC, quality control; ROC, receiver operator characteristic; SCZ, schizophrenia; Sens, sensitivity; Spec, specificity.

Meta-analysis of cohorts 1–5, comprising 127 first-onset drug-naive schizophrenia patients and 204 controls led to initial identification of 29 analytes, which were altered significantly in schizophrenia patients compared with controls (see Supplementary Figure 1 for Forest Plots). This was refined to an optimal set of 26 analytes using the LASSO regression method with 10-fold cross-validation (Table 2). Next, testing of the pooled cohorts 1–5 (discovery metacohort) using the refined 26-analyte panel resulted in excellent performance with an AUC of 0.96 (sensitivity=90%, specificity=90%, accuracy=90%). This was similar to the values obtained with the 29-analyte panel (AUC=0.96, sensitivity=91%, specificity=88%, accuracy=89% Table 3, Figure 2a). The 26 analytes were involved in six main molecular functions: lipid transport (apolipoprotein A1 (ApoA1), apolipoprotein H (ApoH)), inflammation (alpha-2 macroglobulin (A2M), beta-2 microglobulin (B2M), carcinoembryonic antigen (CA), haptoglobin, interleukin-1 receptor antagonist, interleukin-8 (IL8), interleukin-10 (IL10), interleukin-13 (IL13), macrophage migration inhibitory factor (MIF), receptor for advanced glycosylation end products, serum glutamic oxaloacetic transaminase (SGOT), tenascin C (TNC), von Willebrand factor (vWF)), immune system (immunoglobulin A (IgA)), hormonal signalling (follicle-stimulating hormone (FSH), leptin, pancreatic polypeptide (PPP), testosterone, thyroid-stimulating hormone (TSH)), growth factor signalling (AXL receptor tyrosine kinase, insulin-like growth factor-binding protein 2, stem cell factor (SCF)) and the clotting cascade (angiotensin-converting enzyme (ACE), factor VII; Table 2).

### Stage II. Validation of the biomarker panel

The next stage involved validating the diagnostic performance of the 26-analyte panel using samples from two independent European cohorts comprising 93 first-onset schizophrenia patients and 88 controls (cohorts 6 and 7), with similar characteristics to the discovery metacohort (Table 1). For the analysis of cohort 6 (Spain), the assays for CA, IL10, IL13 and SGOT were excluded for failing QC, as described in the Materials and methods. Therefore, a reduced panel of 22 analytes was tested and this yielded an excellent AUC of 0.97 (sensitivity=87%, specificity=97%, accuracy=93% Table 3; Figure 2b). In addition, the full panel was tested on cohort 7 (Germany), which consisted of schizophrenia patients only. For this reason, two classification algorithms (logistic regression and linear discriminant analysis) were trained on the discovery metacohort and tested on cohort 7. This resulted in correct classification (sensitivity) of 89% of the patients (Table 3).

### Stage III. Predictive performance testing of the biomarker panel

For the third phase of the study, the predictive performance of the panel was tested on the pre-schizophrenia/pre-bipolar disorder (USA military, cohort 8) and help-seeker/prodromal (cohort 9) cohorts. All of these individuals were sampled before manifestation of psychotic symptoms as described in the Materials and methods section.

For the testing of cohort 8, assays for A2M and IL10 were excluded for failing QC. This resulted in a final panel of 24 analytes. This cohort comprised 75 pre-schizophrenia and 110 pre-bipolar disorder individuals and 184 healthy controls. Testing of the 24-analyte panel gave an excellent AUC of 0.90 for predicting the development of schizophrenia (sensitivity=88%, specificity=81%, accuracy=85%). We then applied the fitted biomarker model on serum data from 110 pre-bipolar disorder military personnel and controls. This resulted in an AUC of only 0.53 (sensitivity=25%, specificity=86%, accuracy=56% Table 3; Figure 2c) indicating that this algorithm fails to predict the development of bipolar disorder. Further discriminatory performance testing yielded an AUC of 0.91 (sensitivity=88%, specificity=83%, accuracy=85%) for discriminating pre-schizophrenia from pre-bipolar disorder military personnel. These results demonstrated that this biomarker panel has an excellent performance for predicting development of schizophrenia and high differential diagnostic power for discriminating schizophrenia from bipolar disorder patients before disease diagnosis.

For testing of cohort 9, which comprised help-seeking prodromal individuals (18 who later developed schizophrenia and 58 who did not), the assays for CA, IL10, IL13 and SGOT were excluded for failing QC, resulting in a 22-analyte panel (the same panel tested on cohort 6). Testing the predictive performance of this panel resulted in an AUC of 0.82 for prediction of transition to schizophrenia from a prodromal state (sensitivity=89%, specificity=66%, accuracy=71%). We next examined whether this performance could be improved by incorporation of symptom scores into the model. This showed that testing the combination of the 22-analyte panel and CAARMS-positive subscale scores increased the predictive performance to excellent levels with an AUC of 0.90 (sensitivity=89%, specificity=79%, accuracy=82%). In comparison, testing using the CAARMS-positive subscale scores alone led to only a fair predictive performance (AUC=0.72, sensitivity=78%, specificity=60%, accuracy=64% Table 3; Figure 2d).

## Discussion

We and others have previously published on the identification of serum protein biomarkers in schizophrenia patients.^6, 26, 27^ In the present study, we extended these findings by performing a meta-analysis of five independent first- and recent-onset antipsychotic-naive schizophrenia patient cohorts and considered the joint effect of multiple assays in the form of a single biomarker panel for distinguishing patients from controls with excellent performance. We then validated the discriminatory performance of this panel using two independent cohorts. The strength of this study was the demonstration that this biomarker panel had a good predictive performance for identifying individuals who later converted from either a prodromal or an apparently healthy psychological state to schizophrenia. Furthermore, incorporation of symptom scores into the model led to a further increase in performance to excellent levels for prediction of converters in the prodromal cohort.

Recent studies, which have investigated other approaches such as magnetic resonance imaging or psychopathological symptoms have shown a range of diagnostic accuracies ranging from fair to excellent (75–92%) for discriminating schizophrenia patients or pre-onset schizophrenia individuals from controls (for review, see Zarogianni *et al.*^28^). However, most of these studies used relatively small sample sizes. Gene expression profiling studies have also been carried out by other researchers to identify blood-based biomarkers for schizophrenia. For instance, Kurian *et al.*^29^ applied a convergent functional genomics approach to identify blood-based gene expression biomarkers for psychosis. Studies on the use of blood-based microRNAs as diagnostic biomarkers for schizophrenia^30, 31^ have also been carried out, which achieved a range of diagnostic accuracies from fair to good (AUC=0.69–0.85; sensitivity=59–91% specificity=65–81%) for discriminating schizophrenia patients from controls. However, studies aimed at identifying blood-based molecular biomarkers predictive of illness before onset are still rare. Furthermore, the biomarker field for psychiatric disorders is still in its early stages and thus, many studies still lack validation using independent cohorts. This means that, over time, only the most robust findings will survive as more data become available and more extensive validation studies are carried out.^32^

Here, we have identified and validated a protein-based serum biomarker panel for the identification of first-onset schizophrenia patients using seven independent cohorts of patients and controls and showed that the same panel could be used with good-to-excellent diagnostic accuracy for the identification of help-seekers who are at risk of developing a psychiatric illness as well as psychologically healthy individuals who would later transit to schizophrenia using two additional international independent cohorts. Although further validation studies using larger independent pre-onset sample sets are still needed, the current findings may represent the first successful steps in meeting the critical need for early disease detection in psychiatric medicine. Further validation of the schizophrenia biomarker candidates identified here could also lead to new insights into schizophrenia pathophysiology. Several limitations need to be taken into account, which should also form the basis for future studies. We have previously identified gender-specific serum biomarker patterns in both Asperger's syndrome^33^ and schizophrenia^34^ patients. Although no significant gender effect was found in our study, the potential effects of this variable should not be underestimated. Hence, future studies investigating gender-specific markers predictive of transition to schizophrenia are warranted. Similarly, other confounding variables that could potentially influence hormonal regulation and metabolism such as the use of contraceptives, menstrual cycle phase, body mass index and smoking could not be accounted for completely in our analysis as these were either not recorded or only partially recorded (Table 1). Future studies should attempt to account for these factors. Another factor which should be taken into consideration is that all proteins in our study were measured in serum and we can only speculate about their role in the central nervous system. However, we have previously reported that changes in peripheral analyte levels may reflect, at least partly, changes in the brain or vice versa.^35^ This is further supported by evidence implicating systemic influences on brain function involving the immune and metabolic systems in the precipitation and course of psychiatric conditions. These studies indicate that the brain and peripheral systems are intimately connected, which is also reflected in changes in the composition of the blood.^36^ However, it remains a question of debate whether altered brain function is the root cause of peripheral changes or whether, more controversially, peripheral changes precipitate psychiatric symptoms. If this was the case, interventions aimed at normalizing peripheral pathologies associated with mental illness could be indicated. A number of studies suggest that psychiatric symptoms, particularly at early stages of the illness, may be alleviated by targeting affected peripheral pathways such as the immune/inflammatory system. Clinical trials have already shown favourable therapeutic effects of peripheral administration of anti-inflammatory agents such as COX2 inhibitors (for example, Celecoxib).^37^ Finally, we cannot completely rule out the possibility that cohort 8 (military cohort) could be more similar to the first-onset patient/control cohorts 1–7 given the relatively short interval (30 days) between blood collection and initial psychiatric diagnosis. Most of the previous peripheral biomarker studies have examined patients treated with antipsychotic medication, which could have a confounding effect on the circulating analytes. It is difficult to recruit first-onset drug-naive patients as even large psychiatric centres can only recruit around 20–30 of these patients each year, and few centres follow strict standard operating procedures for the collection of samples. We overcame this limitation by including first-onset drug-naive patients from multiple independent clinical centres. Patients were recruited over a period of up to 10 years in specialist early psychosis centres or clinics (see Table 1 for recruitment periods). All the patients and matched controls underwent extensive clinical characterization and sera were collected and stored according to strict standard operating procedures and in compliance with the Standards for Reporting of Diagnostic Accuracy initiative to maximize reliability and accuracy of the results.

The majority of the analytes used in the final test panel are involved in inflammation and immune system functions, consistent with the findings from previous studies.^6, 26, 27^ Effects on inflammation have been widely reported in schizophrenia and appear to involve a mixture of pro- and anti-inflammatory responses (for review, see Miller *et al.*^38^). Previous studies have also reported changes in hormones and growth factors such as chromogranin A, leptin and pancreatic polypeptide.^39^ Other analytes on the panel are involved in lipid transport, hormonal and growth factor signalling and the clotting cascade, in line with the findings of other studies.^6, 40^ Changes in all of these pathways are known to have effects on brain functions such as mood, emotional responses and cognitive processes.^36, 41^ Again, this illustrates how changes in peripheral system can affect central nervous system functions.

Out of our original 29 significant analyte panel, 23 were previously identified in at least one of the four published studies,^6, 7, 26, 27^ which have used a multiplex immunoassay approach to identify blood-based protein biomarkers for schizophrenia. These molecules include A2M, ApoA1, ApoH, CA, eotaxin, factor VII, FSH, HPT, IgA, IGFBP2, IL10, IL1ra, IL13, IL8, leptin, MIF, PPP, SGOT, SCF, testosterone, TSH, VCAM-1 and vWF. However, the overlap with results reported by each individual study was only moderate. The main reason for this is that we conducted a more extensive analysis using very stringent data quality filtration criteria, including exclusion of analytes that failed QC (>30% missing values) and those affected by significant disease-association heterogeneity. This has resulted in the exclusion of many of the analytes reported in previous studies (see Supplementary Table 6 for results overlap with the literature). For instance, in the Schwarz *et al.*^6^ study, 34 molecules were found to be significantly altered in schizophrenia patients relative to controls. Of these, 19 analytes were excluded in our study as they either failed QC (eight analytes) or due to disease-association heterogeneity (11 analytes). So, only 15 significant molecules were measured and analysed in our study. Of these, we found 11 to be significant. Similarly, Schwarz *et al.*^27^ subsequently investigated 53 serum molecules involved in immune response and growth factor signalling and found that based on this profile schizophrenia patients could be separated into two significantly distinct subgroups. Out of the 53 analytes, 37 were excluded in our study (24 failed QC and 13 were affected by disease-association heterogeneity). Only 16 significant molecules were measured in our study, and, of these, we found 10 to be significant.

The results from this and other published findings may also differ depending on whether significant findings are reported based on false discovery rate-adjusted *P*-values or not. For example, in the Domenici *et al.*^26^ study, 10 markers were identified by multivariate analysis of data from schizophrenia patients and controls. Of these, only six analytes were measured in our study, two of which were significant and four were not. From their univariate analysis, 55 analytes were found to be significantly altered in patients based on unadjusted *P*-values<0.05. Of these analytes, 30 were excluded in our study (16 failed QC and 14 showed disease-association heterogeneity). As a result, only 25 significant molecules were also analysed in our study, and, of these, we found 14 to be significant based on false discovery rate-adjusted *P*-values. As Domenici *et al.* presented unadjusted *P*-values, the lack of overlap of the remaining 11 molecules could at least, in part, be due to the lack of control for false discovery rate.

Perkins *et al.*^7^ published the only previous plasma-based protein biomarker study reporting on a 15-analyte panel for predicting schizophrenia conversion. Out of these analytes, 11 were excluded or not measured in our study (three failed QC (IL1 beta, IgE, GH); six were not measured in the Myriad-RBM assay version used in this study (MDA-LDL, MMP-7, uromodulin, Apo D, KIT ligand, chemokine ligand 8); two showed disease-association heterogeneity (cortisol, resistin)). Of the four significant molecules (TSH, factor VII, IL7, IL8) which were measured in our study, we found three to be significant (TSH, Factor VII and IL8). Therefore, the lack of overlap with the findings of Perkins *et al.* is primarily due to the fact that 11 out of 15 analytes (73%) were excluded or not measured in our study, as explained above. Another difference could result from the analysis of different blood substrates as the authors examined plasma and we analysed serum. Furthermore, Perkins *et al.* developed their algorithm through training and testing on the same relatively small cohort of high-risk individuals that did or did not progress to psychosis and controls. In contrast, the samples we used to develop our biomarker panel were obtained from large cohorts of well-characterized first-onset drug-naive schizophrenia patients, who would be expected to show greater homogeneity in their serum molecular profiles with respect to a ‘schizophrenia signal' (that is, the 26-analyte panel). We then demonstrated that this signal was already present at the pre-onset or prodromal stages of the illness as our biomarker panel successfully predicted transition to schizophrenia. It is important to note that we do not imply that analytes that failed the very stringent criteria applied in the present study are not relevant for the schizophrenia disease process or significant in the context of another research question. The aim of our study was to identify the optimal combination of reproducibly measured analytes capable to diagnose and/or predict schizophrenia disease status. Future work attempting to refine biomarker panels should consider testing for those analytes which show the most robust and reproducible measurements across clinical samples to support their utility in the clinic. Finally, the multiplex immunoassay platform used in this study has also been previously applied in studies, which have attempted to identify serum or plasma biomarker profiles in depression and bipolar disorder patients. Out of our 26-analyte panel, only three proteins (ApoH, ApoA1 and B2M) were altered in bipolar disorder patients and four (MIF, ACE, TNC and ILra) were changed in patients with depression. These results suggest that biomarker profiles for these disorders are different, although a direct comparison of protein levels in the same study is required to determine the predictive accuracy of a given diagnostic panel.

Beyond the prognostic and diagnostic potential of the present biomarker panel, these findings may lead to applications for personalized medicine approaches. For example, patients exhibiting changes in inflammation pathways may benefit from anti-inflammatory medication as an adjunctive treatment with standard antipsychotics.^42^ Furthermore, this panel shows promise for future studies aimed at developing a pre-onset differential diagnostic test. We demonstrated that our biomarker panel had a high discriminatory power to differentiate individuals who would later be diagnosed with schizophrenia from those who would later receive a diagnosis of bipolar disorder.

The debates surrounding the prodromal syndrome arise from the lack of diagnostic tools to accurately predict or identify those individuals who will go on to develop schizophrenia (an estimated 20–30% of ultra-high-risk individuals, over a 2–3-year period). This raises ethical issues regarding stigmatization and the potential for inappropriate treatment. All clinical tests have a chance of false diagnosis, which should be considered in the context of a clinical application. In testing the prodromal cohort, we found that the combination of the molecular and symptom-based tests resulted in a higher performance (AUC=0.90) than could be achieved with either test alone. We do not propose that this test should be used to screen the general population, but the data suggest that application of this test in conjunction with currently used structured interviews may aid in earlier and more accurate diagnosis of schizophrenia and thereby facilitate early intervention and improved clinical outcomes.

Market analysis has shown that psychiatrists would value a blood test that could help in the prediction of conversion in prodromal individuals.^43^ The biomarker panel presented here represents a validated set of biomarkers from which a definitive signature for diagnosis and prediction of schizophrenia in the clinical setting could be developed. Ultimately, further developments of the biomarker panel could form the basis of a low-cost blood test, which can complement DSM-5 or ICD-10-based diagnostic approaches. We suggest that the use of such a test in conjunction with a psychiatric assessment will help to position schizophrenia among other biological disorders, such as diabetes and heart disease, ameliorating the stigma and providing hope for better diagnostic and treatment approaches.

## Figures and Tables

**Table 1:** Patient and control baseline characteristics

	*Number*	*Centre (recruitment period)*	*Sex (M/F)*	*Age (years)*	*BMI (kg/m*^*2*^)	*Smoking (Y|N|NR)*	*Cannabis (Y|N|NR)*	*PANSS pos*	*PANSS neg*	*PANSS gen*	*CAARMS pos*
Cohort 1 (106)	52 CT	Mannheim (2000–2009)	27/25	30±8	23±3	20|32|0	30|21|1	NA	NA	NA	NA
	54 SCZ		32/22	30±10	23±4	23|21|0	31|20|3	23±5	24±8	49±10	NA
Cohort 2 (106)	73 CT	Magdeburg (2008–2010)	46/27	32±9	25±4	20|53|0	2|56|15	NA	NA	NA	NA
	33 SCZ		22/11	31±10	24±4	24|9|0	9|24|0	21±6	19±9	43±12	NA
Cohort 3 (39)	23 CT	Magdeburg (2010)	10/13	33±11	23±3	5|18|0	0|23|0	NA	NA	NA	NA
	16 SCZ		8/8	35±11	21±2	6|9|1	0|15|1	19±8	16±4	37±12	NA
Cohort 4 (26)	16 CT	Magdeburg (2010–2011)	8/8	35±11	23±3	1|15|0	0|16|0	NA	NA	NA	NA
	10 SCZ		6/4	37±12	22 ±3	5|5|0	0|10|0	19±8	14±8	33±21	
Cohort 5 (54)	40 CT	Erasmus (2004–2009)	33/7	26±4	NR	NR	NR	NA	NA	NA	NA
	14 SCZ		11/3	24±6	NR	10|4|0	8|6|0	21±3	19±4	35±8	NA
Cohort 6 (135)	88 CT	Santander (2011–2013)	51/37	33±8	26±4	51|37|0	22|66|0	NA	NA	NA	NA
	47 SCZ		28/19	30±9	23±5	24|23|0	20|27|0	24±3^a^	13±6^a^	NR	NA
Cohort 7 (46)	46 SCZ	Muenster (2000–2007)	35/11	27±9	NR	NR	NR	18±7	18±7	NR	NA
Cohort 8: USA military (369)	184 CT	USA DoDSR (1988–2006)	136/48	22±4	NR	NR	NR	NA	NA	NA	NA
	75 Pre-SCZ		67/8	24±5	NR	NR	NR	NR	NR	NR	NA
	110 Pre-BD		70/40	21±4	NR	NR	NR	NR	NR	NR	NA
Cohort 9: help-seeker/prodromal (76)	18 Pre-SCZ	Paris (2009–2013)	11/7	20±3	21±3	9|8|1	7|11|0	16±7	17±7	41±11	13±7
	58 Not pre-SCZ		33/25	22±4	22±4	26|24|8	12|46|0	12±5	15±7	38±10	8±6

Abbreviations: BD, bipolar disorder; BMI, body mass index; gen, general; CAARMS, Comprehensive Assessment of At-Risk Mental State; CT, control; M/F, male/female; N, no; NA, not applicable; neg, negative; NR, not recorded; PANSS, positive and negative syndrome scale; pos, positive; SCZ, schizophrenia; Y, yes.

a Values were obtained via conversion of SAPS and SANS scores.^11^

Values are presented as average±standard deviation.

**Table 2:** Table showing analytes altered in patients compared with controls

*Molecular function*	*Analyte*	*Abbreviation*	*Single analyte effects (logistic regression)*				*Joint analyte effects (LASSO selection)*
			*Coefficient*	*s.e.*	P*-value*	*Adjusted* P*-value*	*Coefficient*
Lipid transport	Apolipoprotein H	ApoH	2.67	1.05	0.011	0.032	2.23
	Apolipoprotein A1	ApoA1	−1.48	0.66	0.026	0.062	−0.31
							
Inflammatory response	Macrophage migration inhibitory factor	MIF	2.89	0.48	1.75E−09	1.56E−07	2.76
	Carcinoembryonic antigen	CA	1.77	0.36	1.13E−06	1.68E−05	1.69
	Tenascin C	TNC	2.89	0.62	3.57E−06	3.97E−05	1.31
	Interleukin-10	IL10	3.55	0.83	1.70E−05	1.51E−04	3.63
	Interleukin-1 receptor antagonist	IL1ra	1.83	0.46	6.27E−05	4.30E−04	0.76
	Receptor for advanced glycosylation end products	RAGE	−2.01	0.52	1.10E−04	7.00E−04	−1.36
	Interleukin-8	IL8	2.30	0.62	2.12E−04	1.25E−03	0.67
	Haptoglobin	HAPT	1.38	0.37	2.30E−04	1.25E−03	1.23
	von Willebrand factor	VWF	1.69	0.56	0.003	0.010	1.66
	Alpha-2 macroglobulin	A2M	3.22	1.07	0.003	0.010	4.79
	Beta-2 microglobulin	B2M	−4.04	1.55	0.009	0.029	−4.59
	Serum glutamic oxaloacetic transaminase	SGOT	1.90	0.83	0.022	0.055	1.67
	Interleukin-13	IL13	1.32	0.67	0.050	0.103	0.19
							
Immune system	Immunoglobulin A	IgA	−1.54	0.63	0.015	0.042	−1.18
							
Hormonal signalling	Pancreatic polypeptide	PPP	1.97	0.34	4.12E−09	1.83E−07	1.80
	Leptin	Leptin	−1.55	0.28	5.42E−08	1.21E−06	−0.69
	Testosterone (total)	TEST	2.08	0.59	4.11E−04	0.002	0.86
	Follicle-stimulating hormone	FSH	1.17	0.34	5.19E−04	0.002	0.33
	Thyroid-stimulating hormone	TSH	−1.19	0.50	0.017	0.047	0.05
							
Growth factor signalling	Insulin-like growth factor-binding protein 2	IGFBP2	2.96	0.62	1.97E−06	2.51E−05	0.33
	AXL receptor tyrosine kinase	AXL	−2.35	0.82	0.004	0.014	−3.93
	Stem cell factor	SCF	−2.20	0.87	0.011	0.032	−1.72
							
Clotting cascade	Factor VII	FVII	−3.92	0.87	6.50E−06	6.43E−05	−2.71
	Angiotensin-converting enzyme	ACE	−1.39	0.67	0.037	0.082	−1.14
							
Hormonal signalling	Chromogranin-A^a^	CGA	0.54	0.24	0.024	0.060	—
							
Growth factor signalling	Vascular cell adhesion molecule-1^a^	VCAM-1	−2.63	1.25	0.036	0.082	—
							
Inflammatory response	Eotaxin^a^	Eotaxin	0.98	0.48	0.041	0.087	—

The analytes are ranked in the order of significance within each molecular function group.

a Not selected by least absolute shrinkage and selection operator (LASSO) regression.

**Table 3:** Assay performance of samples over the three stages of the study

	*AUC (95% CI)*	*FP*	*TP*	*TN*	*FN*	*PPV (%)*	*NPV (%)*	*Sens (%)*	*Spec (%)*	*FPR (%)*	*Acc (%)*
*Discovery metacohort (schizophrenia compared with controls)*											
29-Analyte panel	0.96 (0.938–0.977)	25	116	179	11	82	94	91	88	12	89
Refined 26-analyte panel	0.96 (0.937–0.976)	21	114	183	13	84	93	90	90	10	90
											
*Validation cohorts (schizophrenia compared with controls)*											
Cohort 6	0.97 (0.952–0.996)	3	41	85	6	93	93	87	97	3	93
Cohort 7 (only SCZ)a,^b^	NA	NA	41	NA	5	NA	NA	89	NA	NA	NA
											
*USA military (pre-schizophrenia/pre-bipolar disorder compared with controls)*											
Pre-SCZ vs CT	0.90 (0.856–0.952)	14	66	61	9	82	87	88	81	19	85
Pre-BD^c^ vs CT	0.53 (0.457–0.611)	15	28	94	82	65	53	25	86	14	56
Pre-SCZ vs pre-BD	0.91 (0.865–0.949)	19	66	91	9	78	91	88	83	17	85
											
*Prodromal/help-seeker cohort (individuals who later developed SCZ compared with those who did not)*											
22-Analyte panel	0.82 (0.706–0.925)	20	16	38	2	44	95	89	66	34	71
22-Analyte panel + CAARMS positive	0.90 (0.816–0.978)	12	16	46	2	57	96	89	79	21	82
CAARMS positive	0.72 (0.568–0.865)	23	14	35	4	38	90	78	60	40	64

Abbreviations: Acc, accuracy; AUC, area under curve; BD, bipolar disorder; CAARMS, Comprehensive Assessment of At-Risk Mental State; CT, control; FN, number of false negatives; FP, number of false positives; FPR, false positive rate; NPV, negative predictive value; PPV, positive predictive value; SCZ, schizophrenia; Sens, sensitivity; Spec, specificity; TN, number of true negatives; TP, number of true positives.

a Classification algorithm: logistic regression.

b Linear discriminant analysis (identical results).

c The fitted biomarker model was applied to the serum data from pre-BD individuals and CT to examine its predictive performance to identify BD before disease onset.

Performance of the biomarker testing of all cohorts was evaluated using accuracy, sensitivity, specificity, predictive values, receiver operating characteristic (ROC) curves and area under the ROC curve (AUC: 0.9–1.0=excellent; 0.8–0.9=good; 0.7–0.8=fair; 0.6–0.7=poor; 0.5–0.6=fail). Optimal trade-offs between sensitivity and specificity were determined by maximizing the Youden's index (*J*; calculated by *J*=sensitivity+specificity−1).^25^

## Notes

RJW and RRK are employees of Myriad Genetics. SB and JDC have been consultants for Myriad Genetics until June 2014; SB is the director of Psynova Neurotech. JDC is a consultant for Psynova Neurotech. The remaining authors declare no conflict of interest.
