# Supplementary figures and images for: Development of a blood-based molecular biomarker test for identification of schizophrenia before disease onset
Source: Transl Psychiatry. 2015 Jul 14;5(7):e601–. doi: 10.1038/tp.2015.91 (PMC5068725; doi:10.1038/tp.2015.91)

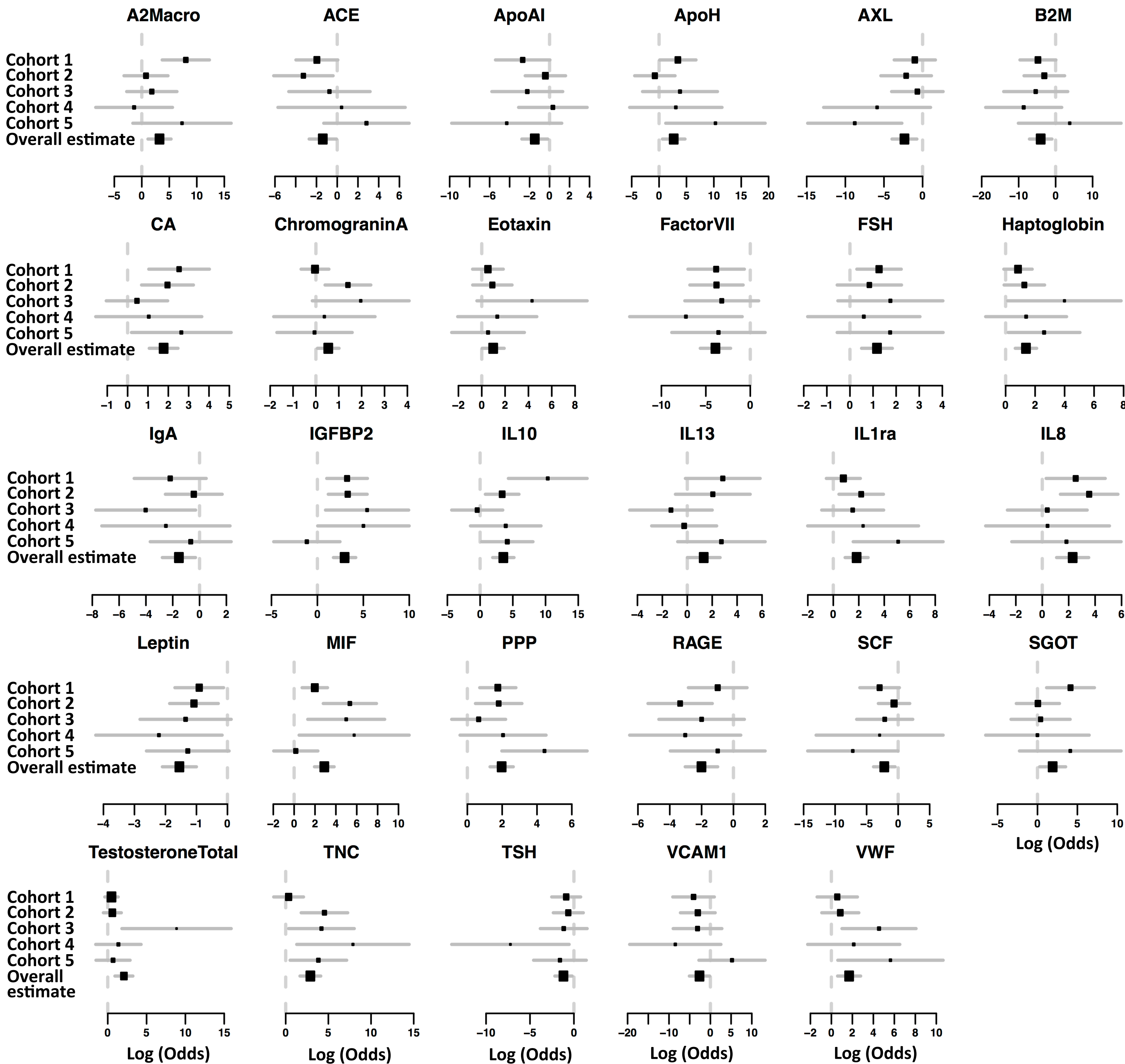

Supplement: Supplementary Figure 1 [file tp201591x1.pdf]
