# Supplementary material for: Development of a blood-based molecular biomarker test for identification of schizophrenia before disease onset
Source: Transl Psychiatry. 2015 Jul 14;5(7):e601–. doi: 10.1038/tp.2015.91 (PMC5068725; doi:10.1038/tp.2015.91)
Supplement: Supplementary Information [file tp201591x2.doc]

**Supplementary information 1. Definition of three subgroups of help-seekers assessed by the CAARMS instrument (cohort 9)**

The CAARMS is a structured interview that encompasses 7 subscales, but only the first subscale (positive symptoms) is used for characterization as UHR or not. This first subscale includes 4 items (unusual thought content, non bizarre ideas, perceptual anomalies, disorganized speech), each separately rated for severity and frequency of symptoms (i.e. a symptom can be subtle but frequent or already severe but unfrequent). The total CAARMS positive subscale was computed as the sum of the mean of ratings of severity and frequency for each of the four items.

The HSCo group were those who presented with psychological distress or psychiatric disorders but did not reach the at-risk mental state criteria. The UHR at-risk group was divided into vulnerability (risk of developing psychosis via a trait risk factor and significant deterioration in mental state and/or functioning), attenuated psychosis (those presenting with a sub-diagnostic threshold or low frequency in recurrence of psychosis) and BLIPS (recent history of psychosis with severity and frequency above the threshold but which disappeared in less than 1 week without antipsychotic treatment) sub-groups. Psychosis was defined by a score of >6 on the CAARMS sub-scale of thought disorder, >5 on the perceptual abnormalities sub-scale and/or >6 on the disorganized speech sub-scale with frequency scores ≥4 on the thought disorder, perceptual abnormalities and/or disorganized speech sub-scales for more than one week.

**Supplementary information 2. Serum sample preparation**

Standard operating protocols were prepared for serum sample preparation and were employed by all clinical centres. In brief, blood samples were collected from all subjects between 8:00 and 12:00 hours into S-Monovette 7.5 mL serum tubes (Sarstedt; Numbrecht, Germany). The blood samples were left to clot at room temperature for 2 hours and then centrifuged at 4000 × g for 5 minutes. The resulting supernatants were stored at −80 °C in Low Binding Eppendorf tubes (Hamburg, Germany).

**Supplementary information 3. Multiplexed immunoassay analyses**

The Multi-Analyte Profiling (MAP) immunoassay platform was used to measure the concentrations of up to 225 analytes in serum samples from the respective institutions (142 in cohorts 1-5 and 7, 187 in the USA military cohort and 225 in cohorts 6 and 9). See **Supplementary Table 1** for details of the percentage of missing values detected in every cohort. These analytes are involved in various hormonal, immune and inflammatory, metabolic and neurotrophic pathways. All the patient samples were randomized and blinded by code numbers until all biochemical assays were completed. All assays were conducted in the Clinical Laboratory Improved Amendments (CLIA)–certified laboratory at Myriad-RBM (Austin, TX, USA) as described previously.^1^ Assays were calibrated using standards and raw intensity measurements were converted to absolute protein concentrations using proprietary software. As described previously^1^, the multiplex immunoassay instrument performance and assay reproducibility were assessed using sets of quality control samples and through reanalysis of the same samples months later. The study protocols, analysis of samples and test methods were carried out in compliance with the Standards for Reporting of Diagnostic Accuracy (STARD) initiative.^2^ The quality control samples had a coefficient of variance below 15%.

**Supplementary information 4. Lasso regression**

Lasso is a penalized method for restricting the residual sum of squares and constraining the sum of the absolute values of the coefficients:
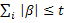
, where *t* is the 'tuning’ parameter. As
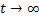
, the effect of *t* is reduced to zero and the solutions are the least squares estimates for the full model. For smaller *t* values, solutions are shrunken versions of the least squares estimates with many coefficients decreased to the null value. *t* was defined using ten-fold cross-validation.

**References**

1. Schwarz E, Guest PC, Rahmoune H, Harris LW, Wang L, Leweke FM *et al.* Identification of a biological signature for schizophrenia in serum. *Mol Psychiatry* 2012; **17**(5)**:** 494-502.

2. Bossuyt PM, Reitsma JB, Bruns DE, Gatsonis CA, Glasziou PP, Irwig LM *et al.* Towards complete and accurate reporting of studies of diagnostic accuracy: the STARD initiative. The Standards for Reporting of Diagnostic Accuracy Group. *Croat Med J* 2003; **44**(5)**:** 635-638.
