# Supplementary material for: Development of a blood-based molecular biomarker test for identification of schizophrenia before disease onset
Source: Transl Psychiatry. 2015 Jul 14;5(7):e601–. doi: 10.1038/tp.2015.91 (PMC5068725; doi:10.1038/tp.2015.91)
Supplement: Supplementary Tables [file tp201591x3.doc]

**Supplementary Table 1. List of all the 142 analytes measured across cohorts 1-5 and 7. For each analyte, the percentages of missing values are shown. Analytes are ordered alphabetically.**

| **Analytes** | **Cohort 1** | **Cohort 2** | **Cohort 3** | **Cohort 4** | **Cohort 5** | **Cohort 7** |
| --- | --- | --- | --- | --- | --- | --- |
| **Angiotensin-Converting Enzyme (ACE)** | 0 | 0 | 0 | 0 | 0 | 0 |
| **ACTH (Adrenocorticotropic Hormone)** | 98 | 70 | 31 | 31 | 63 | 100 |
| **Adiponectin** | 0 | 0 | 0 | 0 | 0 | 0 |
| **Agouti-Related Protein (AgRP)** | 80 | 7 | 56 | 58 | 24 | 85 |
| **Alpha-1-Antitrypsin (AAT)** | 0 | 0 | 0 | 0 | 0 | 0 |
| **Alpha-2-Macroglobulin (A2Macro)** | 0 | 0 | 0 | 0 | 0 | 0 |
| **Alpha-Fetoprotein (AFP)** | 0 | 17 | 5 | 0 | 11 | 2 |
| **Amphiregulin (AR)** | 98 | 33 | 31 | 96 | 57 | 98 |
| **Angiopoietin-2 (ANG-2)** | 1 | 1 | 0 | 0 | 0 | 0 |
| **Angiotensinogen** | 0 | 14 | 0 | 0 | 0 | 0 |
| **Apolipoprotein A-I (Apo A-I)** | 0 | 0 | 0 | 0 | 0 | 0 |
| **Apolipoprotein C-III (Apo C-III)** | 0 | 0 | 0 | 0 | 0 | 0 |
| **Apolipoprotein H (Apo H)** | 0 | 0 | 0 | 0 | 0 | 0 |
| **Apolipoprotein(a) (Lp(a))** | 0 | 0 | 0 | 0 | 0 | 0 |
| **AXL Receptor Tyrosine Kinase (AXL)** | 0 | 0 | 0 | 0 | 0 | 0 |
| **B Lymphocyte Chemoattractant (BLC)** | 21 | 14 | 5 | 4 | 6 | 2 |
| **Brain-Derived Neurotrophic Factor (BDNF)** | 0 | 0 | 0 | 0 | 0 | 0 |
| **Beta-2-Microglobulin (B2M)** | 0 | 0 | 0 | 0 | 0 | 0 |
| **Betacellulin (BTC)** | 48 | 0 | 54 | 54 | 28 | 70 |
| **Bone Morphogenetic Protein 6 (BMP-6)** | 40 | 2 | 3 | 4 | 0 | 85 |
| **C-Reactive Protein (CRP)** | 0 | 0 | 0 | 0 | 0 | 0 |
| **Complement C3** | 0 | 0 | 0 | 0 | 0 | 0 |
| **Calcitonin** | 93 | 52 | 49 | 81 | 59 | 96 |
| **Cancer Antigen 125 (CA-125)** | 42 | 59 | 67 | 0 | 67 | 37 |
| **Cancer Antigen 19-9 (CA-19-9)** | 7 | 8 | 8 | 0 | 17 | 4 |
| **Carcinoembryonic Antigen** | 0 | 6 | 5 | 0 | 0 | 0 |
| **CD 40 antigen (CD40)** | 1 | 0 | 0 | 0 | 0 | 0 |
| **CD40 Ligand (CD40-L)** | 1 | 0 | 0 | 0 | 0 | 0 |
| **Chromogranin-A (CgA)** | 5 | 7 | 0 | 4 | 2 | 0 |
| **Chemokine CC-4 (HCC-4)** | 0 | 0 | 0 | 0 | 0 | 0 |
| **Ciliary Neurotrophic Factor (CNTF)** | 100 | 100 | 100 | 100 | 100 | 100 |
| **Connective Tissue Growth Factor (CTGF)** | 0 | 69 | 26 | 96 | 2 | 0 |
| **Cortisol (Cortisol)** | 0 | 0 | 0 | 0 | 0 | 0 |
| **Creatine Kinase-MB (CK-MB)** | 0 | 0 | 0 | 4 | 2 | 0 |
| **Epidermal Growth Factor (EGF)** | 6 | 0 | 0 | 0 | 0 | 0 |
| **Epithelial-Derived Neutrophil-Activating Protein 78 (ENA-78)** | 0 | 0 | 0 | 0 | 0 | 0 |
| **Endothelin-1 (ET-1)** | 75 | 94 | 97 | 100 | 100 | 70 |
| **EN-RAGE** | 0 | 0 | 0 | 0 | 0 | 0 |
| **Eotaxin** | 2 | 0 | 0 | 0 | 0 | 0 |
| **Eotaxin-3** | 40 | 95 | 100 | 96 | 74 | 41 |
| **Epiregulin (EPR)** | 100 | 49 | 51 | 92 | 98 | 98 |
| **Erythropoietin (EPO)** | 58 | 19 | 51 | 65 | 63 | 57 |
| **FABP** | 24 | 42 | 23 | 15 | 43 | 4 |
| **Factor VII** | 0 | 0 | 0 | 0 | 0 | 0 |
| **FASLG Receptor (FAS)** | 0 | 0 | 3 | 0 | 0 | 0 |
| **Fas Ligand (FasL)** | 32 | 1 | 3 | 0 | 0 | 52 |
| **Ferritin (FRTN)** | 0 | 0 | 0 | 0 | 0 | 0 |
| **Fibroblast Growth Factor basic (FGF-basic)** | 8 | 72 | 79 | 58 | 80 | 2 |
| **Fibroblast Growth Factor 4 (FGF-4)** | 98 | 82 | 36 | 15 | 93 | 98 |
| **Fibrinogen** | 21 | 61 | 56 | 65 | 52 | 26 |
| **Follicle-Stimulating Hormone (FSH)** | 6 | 0 | 0 | 0 | 4 | 9 |
| **Granulocyte Colony-Stimulating Factor (G-CSF)** | 8 | 4 | 8 | 38 | 2 | 11 |
| **Growth Hormone (GH)** | 4 | 13 | 10 | 4 | 37 | 0 |
| **Glucagon-like Peptide 1, total (GLP-1 total)** | 92 | 39 | 5 | 4 | 89 | 100 |
| **Glucagon** | 99 | 99 | 100 | 88 | 89 | 100 |
| **Glutathione S-Transferase alpha (GST-alpha)** | 0 | 4 | 3 | 12 | 7 | 0 |
| **Granulocyte-Macrophage Colony-Stimulating Factor (GM-CSF)** | 65 | 82 | 72 | 88 | 76 | 61 |
| **GRO-alpha** | 0 | 0 | 0 | 0 | 0 | 0 |
| **Haptoglobin** | 1 | 1 | 0 | 0 | 0 | 2 |
| **Heparin-Binding EGF-Like Growth Factor (HB-EGF)** | 0 | 0 | 0 | 0 | 0 | 0 |
| **Hepatocyte Growth Factor (HGF)** | 0 | 0 | 3 | 0 | 0 | 0 |
| **T Lymphocyte-Secreted Protein I-309 (I-309)** | 38 | 4 | 8 | 12 | 7 | 37 |
| **Intercellular Adhesion Molecule 1 (ICAM-1)** | 0 | 0 | 0 | 0 | 0 | 0 |
| **Interferon gamma (IFN-gamma)** | 75 | 29 | 23 | 65 | 26 | 67 |
| **Immunoglobulin A (IgA)** | 0 | 0 | 0 | 0 | 0 | 0 |
| **Immunoglobulin E (IgE)** | 12 | 25 | 28 | 31 | 7 | 4 |
| **Insulin-like Growth Factor-Binding Protein 2 (IGFBP-2)** | 0 | 0 | 0 | 0 | 0 | 0 |
| **Immunoglobulin M (IgM)** | 0 | 0 | 0 | 0 | 0 | 0 |
| **Interleukin-10 (IL-10)** | 0 | 0 | 0 | 0 | 4 | 0 |
| **IL-11 (Interleukin-11)** | 100 | 99 | 92 | 88 | 93 | 100 |
| **Interleukin-12 Subunit p70 (IL-12p70)** | 0 | 100 | 100 | 96 | 89 | 0 |
| **Interleukin-13 (IL-13)** | 5 | 4 | 0 | 4 | 0 | 0 |
| **Interleukin-15 (IL-15)** | 0 | 12 | 18 | 62 | 2 | 0 |
| **Interleukin-16 (IL-16)** | 0 | 0 | 0 | 0 | 0 | 0 |
| **Interleukin-18 (IL-18)** | 0 | 0 | 0 | 0 | 0 | 0 |
| **Interleukin-1 alpha (IL-1 alpha)** | 85 | 3 | 3 | 81 | 54 | 78 |
| **Interleukin-1 beta (IL-1 beta)** | 12 | 82 | 79 | 35 | 96 | 7 |
| **Interleukin-1 receptor antagonist (IL-1ra)** | 2 | 0 | 0 | 0 | 0 | 4 |
| **Interleukin-2 (IL-2)** | 98 | 43 | 56 | 73 | 91 | 100 |
| **Interleukin-3 (IL-3)** | 22 | 1 | 0 | 0 | 2 | 7 |
| **Interleukin-4 (IL-4)** | 2 | 98 | 100 | 100 | 59 | 7 |
| **Interleukin-5 (IL-5)** | 22 | 11 | 28 | 12 | 13 | 20 |
| **Interleukin-6 (IL-6)** | 91 | 74 | 85 | 62 | 76 | 67 |
| **Interleukin-7 (IL-7)** | 0 | 4 | 10 | 15 | 2 | 0 |
| **Interleukin-8 (IL-8)** | 1 | 0 | 0 | 0 | 0 | 2 |
| **Insulin** | 1 | 0 | 0 | 0 | 0 | 4 |
| **Interleukin-12 Subunit p40 (IL-12p40)** | 75 | 16 | 5 | 27 | 17 | 83 |
| **Leptin** | 0 | 0 | 0 | 0 | 0 | 0 |
| **Luteinizing Hormone (LH)** | 4 | 1 | 0 | 0 | 31 | 4 |
| **Lymphotactin** | 96 | 96 | 85 | 77 | 87 | 96 |
| **Macrophage Colony-Stimulating Factor 1 (M-CSF)** | 100 | 8 | 0 | 35 | 100 | 100 |
| **Macrophage Inflammatory Protein-1 alpha (MIP-1 alpha)** | 0 | 0 | 0 | 0 | 0 | 0 |
| **Macrophage Inflammatory Protein-1 beta (MIP-1 beta)** | 0 | 0 | 0 | 0 | 0 | 0 |
| **Macrophage Migration Inhibitory Factor (MIF)** | 1 | 1 | 0 | 0 | 0 | 0 |
| **Macrophage-Derived Chemokine (MDC)** | 0 | 0 | 0 | 0 | 0 | 0 |
| **Matrix Metalloproteinase-2 (MMP-2)** | 76 | 34 | 5 | 0 | 81 | 91 |
| **Matrix Metalloproteinase-3 (MMP-3)** | 0 | 0 | 0 | 0 | 0 | 0 |
| **Matrix Metalloproteinase-9 (MMP-9)** | 86 | 88 | 77 | 50 | 100 | 80 |
| **Monocyte Chemotactic Protein 1 (MCP-1)** | 0 | 0 | 0 | 0 | 0 | 0 |
| **Monocyte Chemotactic Protein 3 (MCP-3)** | 100 | 98 | 90 | 96 | 94 | 98 |
| **Myeloperoxidase (MPO)** | 0 | 0 | 0 | 0 | 0 | 0 |
| **Myoglobin** | 0 | 0 | 0 | 0 | 0 | 0 |
| **Nerve Growth Factor beta (NGF-beta)** | 99 | 89 | 95 | 96 | 98 | 100 |
| **Neuronal Cell Adhesion Molecule (Nr-CAM)** | 2 | 92 | 97 | 81 | 30 | 11 |
| **Plasminogen Activator Inhibitor 1 (PAI-1)** | 0 | 0 | 0 | 0 | 0 | 0 |
| **Pancreatic Polypeptide (PPP)** | 0 | 0 | 0 | 0 | 0 | 0 |
| **PAPP-A** | 93 | 73 | 79 | 54 | 76 | 89 |
| **Pulmonary and Activation-Regulated Chemokine (PARC)** | 0 | 0 | 0 | 0 | 0 | 0 |
| **PDGF** | 1 | 0 | 0 | 0 | 0 | 0 |
| **Peptide YY (PYY)** | 88 | 56 | 51 | 54 | 69 | 89 |
| **Progesterone** | 0 | 3 | 0 | 0 | 0 | 2 |
| **Prolactin (PRL)** | 0 | 0 | 0 | 0 | 0 | 0 |
| **Prostate-Specific Antigen, Free (PSA-f)** | 45 | 34 | 46 | 46 | 19 | 17 |
| **Prostatic Acid Phosphatase (PAP)** | 0 | 0 | 0 | 4 | 0 | 0 |
| **T-Cell-Specific Protein RANTES (RANTES)** | 0 | 0 | 0 | 0 | 0 | 0 |
| **Receptor for advanced glycosylation end products (RAGE)** | 0 | 0 | 0 | 0 | 0 | 0 |
| **Resistin** | 0 | 0 | 0 | 0 | 0 | 0 |
| **S100 calcium-binding protein B (S100-B)** | 99 | 25 | 33 | 35 | 13 | 100 |
| **Serum Amyloid P-Component (SAP)** | 0 | 0 | 0 | 0 | 0 | 0 |
| **Stem Cell Factor (SCF)** | 0 | 0 | 0 | 0 | 0 | 0 |
| **Secretin** | 94 | 99 | 97 | 100 | 96 | 91 |
| **Serum Glutamic Oxaloacetic Transaminase (SGOT)** | 0 | 0 | 0 | 0 | 0 | 0 |
| **Sex Hormone-Binding Globulin (SHBG)** | 0 | 0 | 0 | 0 | 0 | 0 |
| **SOD** | 0 | 0 | 0 | 0 | 0 | 0 |
| **Sortilin** | 0 | 0 | 0 | 0 | 0 | 0 |
| **Tenascin-C (TN-C)** | 0 | 0 | 0 | 0 | 0 | 0 |
| **Testosterone, Total** | 0 | 1 | 0 | 0 | 0 | 0 |
| **Transforming Growth Factor alpha (TGF-alpha)** | 88 | 48 | 10 | 50 | 74 | 89 |
| **Transforming Growth Factor beta-3 (TGF-beta-3)** | 97 | 88 | 95 | 96 | 93 | 100 |
| **Thrombopoietin (TPO)** | 0 | 0 | 0 | 0 | 0 | 0 |
| **Thrombospondin-1** | 0 | 0 | 0 | 0 | 0 | 0 |
| **Thyroid-Stimulating Hormone (TSH)** | 0 | 0 | 0 | 0 | 0 | 0 |
| **Thyroxine-Binding Globulin (TBG)** | 0 | 0 | 0 | 0 | 0 | 0 |
| **Tissue Inhibitor of Metalloproteinases 1 (TIMP-1)** | 0 | 0 | 0 | 0 | 0 | 0 |
| **Tissue Factor (TF)** | 3 | 96 | 92 | 96 | 100 | 2 |
| **Tumor necrosis factor receptor 2 (TNFR2)** | 0 | 0 | 0 | 0 | 0 | 0 |
| **Tumor Necrosis Factor alpha (TNF-alpha)** | 1 | 29 | 10 | 0 | 46 | 2 |
| **Tumor Necrosis Factor beta (TNF-beta)** | 90 | 31 | 13 | 35 | 72 | 85 |
| **TNF-Related Apoptosis-Inducing Ligand Receptor 3 (TRAIL-R3)** | 0 | 0 | 0 | 0 | 0 | 0 |
| **Vascular Cell Adhesion Molecule-1 (VCAM-1)** | 0 | 0 | 0 | 0 | 0 | 0 |
| **Vascular Endothelial Growth Factor (VEGF)** | 0 | 0 | 0 | 0 | 0 | 0 |
| **von Willebrand Factor (vWF)** | 0 | 0 | 0 | 0 | 0 | 0 |

**Supplementary Table 2. The list of 89 analytes detected in over 70% of samples and across all five cohorts (1-5) pooled for meta-analysis. This list of analytes was obtained after exclusion of 53 analytes which failed QC out of the original 142 analytes measured, as shown in Supplementary Figure 1. Analytes are listed in alphabetical order.**

| Adiponectin | Interleukin-16 |
| --- | --- |
| Alpha-1 Antitrypsin | Interleukin-18 |
| Alpha-2 Macroglobulin | Interleukin-3 |
| Alpha-Fetoprotein | Interleukin-5 |
| Angiopoietin-2 | Interleukin-7 |
| Angiotensin-Converting Enzyme | Interleukin-8 |
| Angiotensinogen | Leptin |
| Apolipoprotein A1 | Lipoprotein (a) |
| Apolipoprotein CIII | Macrophage Inflammatory Protein-1 alpha |
| Apolipoprotein H | Macrophage Inflammatory Protein-1 beta |
| AXL Receptor Tyrosine Kinase | Macrophage Migration Inhibitory Factor |
| Beta-2 Microglobulin | Macrophage-Derived Chemokine |
| B-Lymphocyte Chemoattractant | Matrix Metalloproteinase-3 |
| Brain-Derived Neurotrophic Factor | Monocyte Chemotactic Protein 1 |
| C Reactive Protein | Myeloperoxidase |
| Cancer Antigen 19-9 | Myoglobin |
| Carcinoembryonic Antigen | Pancreatic polypeptide |
| CD 40 antigen | Plasminogen Activator Inhibitor 1 |
| CD40 Ligand | Platelet-Derived Growth Factor BB |
| Chemokine CC-4 | Progesterone |
| Chromogranin-A | Prolactin |
| Complement C3 | Prostatic Acid Phosphatase |
| Cortisol | Pulmonary and Activation-Regulated Chemokine |
| Creatine Kinase-MB | Receptor for advanced glycosylation end products |
| EN-RAGE | Resistin |
| Eotaxin | Serum Amyloid P-Component |
| Epidermal Growth Factor | Serum Glutamic Oxaloacetic Transaminase |
| Epithelial-Derived Neutrophil-Activating Protein 78 | Sex Hormone-Binding Globulin |
| Factor VII | Sortilin |
| FASLG Receptor | Stem Cell Factor |
| Ferritin | Superoxide Dismutase 1, Soluble |
| Follicle-Stimulating Hormone | T-Cell-Specific Protein RANTES |
| Glutathione S-Transferase alpha | Tenascin C |
| Growth-Regulated alpha protein | Testosterone |
| Haptoglobin | Thrombopoietin |
| Heparin-Binding EGF-Like Growth Factor | Thrombospondin-1 |
| Hepatocyte Growth Factor | Thyroid Stimulating Hormone |
| Immunoglobulin A | Thyroxine-Binding Globulin |
| Immunoglobulin M | Tissue Inhibitor of Metalloproteinases 1 |
| Insulin | TNF-Related Apoptosis-Inducing Ligand Receptor 3 |
| Insulin-like Growth Factor-Binding Protein 2 | Tumor Necrosis Factor Receptor-Like 2 |
| Intercellular Adhesion Molecule 1 | Vascular Cell Adhesion Molecule-1 |
| Interleukin-1 receptor antagonist | Vascular Endothelial Growth Factor |
| Interleukin-10 | von Willebrand Factor |
| Interleukin-13 |  |

**Supplementary Table 3. The list of 27 analytes affected by disease association heterogeneity. Analytes are listed in alphabetical order.**

| **Analyte** | **Abbrev.** | **Coefficient** | **Std. Error** | **P-value** |
| --- | --- | --- | --- | --- |
| **Alpha-1 Antitrypsin** | **AAT** | 4.04 | 1.11 | 0.028 |
| **Angiopoietin-2** | **Angiopoietin2** | 1.24 | 0.47 | 0.022 |
| **Angiotensinogen** | **Angiotensinogen** | 0.23 | 0.15 | 1.84E-04 |
| **Apolipoprotein CIII** | **ApoCIII** | -3.15 | 0.76 | 0.019 |
| **Brain-Derived Neurotrophic Factor** | **BDNF** | -1.58 | 0.92 | 0.026 |
| **C Reactive Protein** | **CRP** | -0.41 | 0.19 | 0.030 |
| **Cortisol** | **Cortisol** | 2.52 | 0.63 | 0.025 |
| **EN-RAGE** | **ENRAGE** | 2.07 | 0.37 | 4.89E-06 |
| **Epidermal Growth Factor** | **EGF** | 0.08 | 0.37 | 7.97E-10 |
| **Ferritin** | **FRTN** | 1.53 | 0.30 | 0.002 |
| **Glutathione S-Transferase alpha** | **GSTalpha** | 0.31 | 0.31 | 1.13E-06 |
| **Insulin** | **Insulin** | 0.67 | 0.29 | 0.012 |
| **Intercellular Adhesion Molecule 1** | **ICAM1** | 0.23 | 1.08 | 7.26E-04 |
| **Interleukin-16** | **IL16** | 2.27 | 0.79 | 9.82E-05 |
| **Interleukin-5** | **IL5** | -0.68 | 0.33 | 0.038 |
| **Myeloperoxidase** | **Myeloperoxidase** | 1.23 | 0.43 | 5.98E-04 |
| **Progesterone** | **Progesterone** | 1.76 | 0.53 | 2.24E-04 |
| **Prolactin** | **PRL** | 0.63 | 0.36 | 0.008 |
| **Resistin** | **Resistin** | 0.86 | 0.62 | 3.54E-04 |
| **Sortilin** | **Sortilin** | -1.40 | 0.87 | 3.49E-05 |
| **Superoxide Dismutase 1, Soluble** | **SOD** | 2.08 | 0.57 | 0.022 |
| **Thrombopoietin** | **TPO** | -0.46 | 0.85 | 0.014 |
| **Thyroxine-Binding Globulin** | **TBG** | 0.78 | 1.03 | 0.020 |
| **Tissue Inhibitor of Metalloproteinases 1** | **TIMP1** | 2.48 | 1.42 | 0.027 |
| **TNF-Related Apoptosis-Inducing Ligand Receptor 3** | **TRAILR3** | 2.07 | 0.62 | 0.023 |
| **Tumor Necrosis Factor Receptor-Like 2** | **TNFRII** | 0.87 | 1.19 | 0.014 |
| **Vascular Endothelial Growth Factor** | **VEGF** | -0.75 | 0.71 | 0.009 |

P-value shows significance of the disease association heterogeneity test. P-value<0.05 indicates that the respective analyte is significantly affected by disease association heterogeneity. To test for this association, linear regression analyses were carried out examining interaction between cohort and diagnosis on the analyte levels (outcome).

**Supplementary Table 4. List of all the 225 analytes measured in cohorts 6 and 9. These cohorts were run in one experiment and samples were randomised to avoid experimental bias. For each analyte, the percentages of missing values are shown. Analytes are listed in alphabetical order.**

| **Analyte** | **Cohort 6** | **Cohort 9** |
| --- | --- | --- |
| **6Ckine** | 0 | 0 |
| **Adiponectin** | 0 | 0 |
| **Agouti-Related Protein (AgRP)** | 100 | 100 |
| **Alpha-1-acid glycoprotein 1 ( AGP-1)** | 0 | 0 |
| **Alpha-1-Antichymotrypsin (AACT)** | 0 | 0 |
| **Alpha-1-Antitrypsin (AAT)** | 0 | 0 |
| **Alpha-1-Microglobulin (A1Micro)** | 0 | 0 |
| **Alpha-2-Macroglobulin (A2Macro)** | 0 | 0 |
| **Amphiregulin (AR)** | 3 | 3 |
| **Angiogenin** | 0 | 0 |
| **Angiopoietin-2 (ANG-2)** | 0 | 0 |
| **Angiotensin-Converting Enzyme (ACE)** | 0 | 0 |
| **Antithrombin-III (AT-III)** | 0 | 0 |
| **Apolipoprotein A-I (Apo A-I)** | 0 | 0 |
| **Apolipoprotein A-II (Apo A-II)** | 0 | 0 |
| **Apolipoprotein A-IV (Apo A-IV)** | 0 | 0 |
| **Apolipoprotein B (Apo B)** | 0 | 0 |
| **Apolipoprotein C-I (Apo C-I)** | 0 | 0 |
| **Apolipoprotein C-III (Apo C-III)** | 0 | 0 |
| **Apolipoprotein D (Apo D)** | 3 | 3 |
| **Apolipoprotein E (Apo E)** | 0 | 0 |
| **Apolipoprotein H (Apo H)** | 0 | 0 |
| **Apolipoprotein(a) (Lp(a))** | 0 | 0 |
| **AXL Receptor Tyrosine Kinase (AXL)** | 0 | 0 |
| **B cell-activating factor (BAFF)** | 0 | 0 |
| **B Lymphocyte Chemoattractant (BLC)** | 97 | 97 |
| **Beta-2-Microglobulin (B2M)** | 0 | 0 |
| **Betacellulin (BTC)** | 100 | 100 |
| **Brain-Derived Neurotrophic Factor (BDNF)** | 0 | 0 |
| **Calbindin** | 100 | 100 |
| **Cancer Antigen 15-3 (CA-15-3)** | 0 | 0 |
| **Cancer Antigen 72-4 (CA 72-4)** | 79 | 79 |
| **Cartilage Oligomeric Matrix Protein (COMP)** | 0 | 0 |
| **Cathepsin D** | 0 | 0 |
| **CD 40 antigen (CD40)** | 0 | 0 |
| **CD40 Ligand (CD40-L)** | 0 | 0 |
| **CD5 Antigen-like (CD5L)** | 0 | 0 |
| **Chemokine CC-4 (HCC-4)** | 0 | 0 |
| **Chromogranin-A (CgA)** | 0 | 0 |
| **Ciliary Neurotrophic Factor (CNTF)** | 100 | 100 |
| **Clusterin (CLU)** | 0 | 0 |
| **Collagen IV** | 0 | 0 |
| **Complement C3 (C3)** | 0 | 0 |
| **Complement component C1q receptor (C1qR1)** | 0 | 0 |
| **Complement Factor H – Related Protein 1 (CFHR1)** | 0 | 0 |
| **Cortisol (Cortisol)** | 0 | 0 |
| **C-Peptide** | 0 | 0 |
| **C-Reactive Protein (CRP)** | 13 | 13 |
| **Creatine Kinase-MB (CK-MB)** | 2 | 2 |
| **Cystatin-C** | 0 | 0 |
| **Dipeptidyl peptidase IV (DPPIV)** | 0 | 0 |
| **Endoglin** | 0 | 0 |
| **EN-RAGE** | 0 | 0 |
| **Eotaxin-1** | 7 | 7 |
| **Eotaxin-2** | 0 | 0 |
| **Eotaxin-3** | 99 | 99 |
| **Epidermal Growth Factor (EGF)** | 0 | 0 |
| **Epidermal Growth Factor Receptor (EGFR)** | 0 | 0 |
| **Epiregulin (EPR)** | 1 | 1 |
| **Epithelial-Derived Neutrophil-Activating Protein 78 (ENA-78)** | 0 | 0 |
| **E-Selectin** | 0 | 0 |
| **Factor VII** | 0 | 0 |
| **Fas Ligand (FasL)** | 94 | 94 |
| **FASLG Receptor (FAS)** | 0 | 0 |
| **Fatty Acid-Binding Protein, adipocyte (FABP, adipocyte)** | 0 | 0 |
| **Fatty Acid-Binding Protein, liver (FABP, liver)** | 88 | 88 |
| **Ferritin (FRTN)** | 0 | 0 |
| **Fetuin-A** | 0 | 0 |
| **Fibrinogen** | 100 | 100 |
| **Fibroblast Growth Factor 4 (FGF-4)** | 100 | 100 |
| **Fibroblast Growth Factor basic (FGF-basic)** | 19 | 19 |
| **Fibulin-1C (Fib-1C)** | 0 | 0 |
| **Ficolin-3** | 0 | 0 |
| **Follicle-Stimulating Hormone (FSH)** | 0 | 0 |
| **Galectin-3** | 0 | 0 |
| **Gelsolin** | 0 | 0 |
| **Glucagon-like Peptide 1, active (GLP-1 active)** | 100 | 100 |
| **Glucagon-like Peptide 1, total (GLP-1 total)** | 91 | 91 |
| **Granulocyte Colony-Stimulating Factor (G-CSF)** | 37 | 37 |
| **Granulocyte-Macrophage Colony-Stimulating Factor (GM-CSF)** | 100 | 100 |
| **Growth Hormone (GH)** | 21 | 21 |
| **Haptoglobin** | 1 | 1 |
| **Heat Shock Protein 60 (HSP-60)** | 98 | 98 |
| **Hemopexin** | 0 | 0 |
| **Heparin-Binding EGF-Like Growth Factor (HB-EGF)** | 38 | 38 |
| **Hepatocyte Growth Factor (HGF)** | 0 | 0 |
| **Hepatocyte Growth Factor receptor (HGF receptor)** | 0 | 0 |
| **Hepsin** | 0 | 0 |
| **Human Epidermal Growth Factor Receptor 2 (HER-2)** | 0 | 0 |
| **Immunoglobulin A (IgA)** | 0 | 0 |
| **Immunoglobulin E (IgE)** | 29 | 29 |
| **Immunoglobulin M (IgM)** | 0 | 0 |
| **Insulin** | 28 | 28 |
| **Insulin-like Growth Factor Binding Protein 4 (IGFBP4)** | 0 | 0 |
| **Insulin-like Growth Factor Binding Protein 5 (IGFBP5)** | 0 | 0 |
| **Insulin-like Growth Factor Binding Protein 6 (IGFBP6)** | 0 | 0 |
| **Insulin-like Growth Factor-Binding Protein 1 (IGFBP-1)** | 2 | 2 |
| **Insulin-like Growth Factor-Binding Protein 2 (IGFBP-2)** | 0 | 0 |
| **Insulin-like Growth Factor-Binding Protein 3 (IGFBP-3)** | 0 | 0 |
| **Intercellular Adhesion Molecule 1 (ICAM-1)** | 0 | 0 |
| **Interferon gamma (IFN-gamma)** | 100 | 100 |
| **Interferon gamma Induced Protein 10 (IP-10)** | 0 | 0 |
| **Interferon-inducible T-cell alpha chemoattractant (ITAC)** | 9 | 9 |
| **Interleukin-1 alpha (IL-1 alpha)** | 100 | 100 |
| **Interleukin-1 beta (IL-1 beta)** | 33 | 33 |
| **Interleukin-1 receptor antagonist (IL-1ra)** | 8 | 8 |
| **Interleukin-10 (IL-10)** | 80 | 80 |
| **Interleukin-12 Subunit p40 (IL-12p40)** | 18 | 18 |
| **Interleukin-12 Subunit p70 (IL-12p70)** | 100 | 100 |
| **Interleukin-13 (IL-13)** | 100 | 100 |
| **Interleukin-15 (IL-15)** | 98 | 98 |
| **Interleukin-16 (IL-16)** | 0 | 0 |
| **Interleukin-17 (IL-17)** | 83 | 83 |
| **Interleukin-18 (IL-18)** | 0 | 0 |
| **Interleukin-2 (IL-2)** | 100 | 100 |
| **Interleukin-2 receptor alpha (IL-2 receptor alpha)** | 0 | 0 |
| **Interleukin-23 (IL-23)** | 24 | 24 |
| **Interleukin-3 (IL-3)** | 100 | 100 |
| **Interleukin-4 (IL-4)** | 100 | 100 |
| **Interleukin-5 (IL-5)** | 100 | 100 |
| **Interleukin-6 (IL-6)** | 98 | 98 |
| **Interleukin-6 receptor (IL-6r)** | 0 | 0 |
| **Interleukin-6 receptor subunit beta (IL-6R beta)** | 0 | 0 |
| **Interleukin-7 (IL-7)** | 100 | 100 |
| **Interleukin-8 (IL-8)** | 27 | 27 |
| **Kallikrein 5** | 0 | 0 |
| **Kidney Injury Molecule-1 (KIM-1)** | 98 | 98 |
| **Latency-Associated Peptide of Transforming Growth Factor beta 1 (LAP TGF-b1)** | 0 | 0 |
| **Leptin** | 0 | 0 |
| **Leucine-rich alpha-2-glycoprotein (LRG1)** | 0 | 0 |
| **Lumican** | 0 | 0 |
| **Luteinizing Hormone (LH)** | 20 | 20 |
| **Macrophage Colony-Stimulating Factor 1 (M-CSF)** | 14 | 14 |
| **Macrophage inflammatory protein 3 beta (MIP-3 beta)** | 0 | 0 |
| **Macrophage Inflammatory Protein-1 alpha (MIP-1 alpha)** | 93 | 93 |
| **Macrophage Inflammatory Protein-1 beta (MIP-1 beta)** | 0 | 0 |
| **Macrophage Inflammatory Protein-3 alpha (MIP-3 alpha)** | 80 | 80 |
| **Macrophage Migration Inhibitory Factor (MIF)** | 2 | 2 |
| **Macrophage-Derived Chemokine (MDC)** | 0 | 0 |
| **Macrophage-Stimulating Protein (MSP)** | 0 | 0 |
| **Matrix Metalloproteinase-1 (MMP-1)** | 2 | 2 |
| **Matrix Metalloproteinase-10 (MMP-10)** | 0 | 0 |
| **Matrix Metalloproteinase-3 (MMP-3)** | 0 | 0 |
| **Matrix Metalloproteinase-7 (MMP-7)** | 0 | 0 |
| **Matrix Metalloproteinase-9 (MMP-9)** | 39 | 39 |
| **Matrix Metalloproteinase-9, total (MMP-9, total)** | 0 | 0 |
| **MHC class I chain-related protein A (MICA)** | 71 | 71 |
| **Monocyte Chemotactic Protein 1 (MCP-1)** | 0 | 0 |
| **Monocyte Chemotactic Protein 2 (MCP-2)** | 1 | 1 |
| **Monocyte Chemotactic Protein 3 (MCP-3)** | 100 | 100 |
| **Monocyte Chemotactic Protein 4 (MCP-4)** | 0 | 0 |
| **Monokine Induced by Gamma Interferon (MIG)** | 5 | 5 |
| **Myeloid Progenitor Inhibitory Factor 1 (MPIF-1)** | 0 | 0 |
| **Myeloperoxidase (MPO)** | 1 | 1 |
| **Myoglobin** | 0 | 0 |
| **Nerve Growth Factor beta (NGF-beta)** | 100 | 100 |
| **Neuronal Cell Adhesion Molecule (Nr-CAM)** | 3 | 3 |
| **Neuropilin-1** | 0 | 0 |
| **Neutrophil Activating Peptide 2 (NAP-2)** | 0 | 0 |
| **Neutrophil Gelatinase-Associated Lipocalin (NGAL)** | 0 | 0 |
| **Osteopontin** | 0 | 0 |
| **Osteoprotegerin (OPG)** | 0 | 0 |
| **Pancreatic Polypeptide (PPP)** | 0 | 0 |
| **Pepsinogen I (PGI)** | 0 | 0 |
| **Peptidase D (PEPD)** | 0 | 0 |
| **Pigment Epithelium Derived Factor (PEDF)** | 0 | 0 |
| **Placenta Growth Factor (PLGF)** | 3 | 3 |
| **Plasminogen Activator Inhibitor 1 (PAI-1)** | 0 | 0 |
| **Platelet-Derived Growth Factor BB (PDGF-BB)** | 0 | 0 |
| **Progesterone** | 0 | 0 |
| **Proinsulin, Intact** | 100 | 100 |
| **Proinsulin, Total** | 100 | 100 |
| **Prolactin (PRL)** | 0 | 0 |
| **Prostate-Specific Antigen, Free (PSA-f)** | 40 | 40 |
| **Pulmonary and Activation-Regulated Chemokine (PARC)** | 0 | 0 |
| **Receptor for advanced glycosylation end products (RAGE)** | 0 | 0 |
| **Receptor tyrosine-protein kinase erbB-3 (ErbB3)** | 0 | 0 |
| **Resistin** | 0 | 0 |
| **Retinol-binding protein 4 (RBP-4)** | 0 | 0 |
| **S100 calcium-binding protein B (S100-B)** | 100 | 100 |
| **Serotransferrin (Transferrin)** | 0 | 0 |
| **Serum Amyloid A Protein (SAA)** | 0 | 0 |
| **Serum Amyloid P-Component (SAP)** | 0 | 0 |
| **Sex Hormone-Binding Globulin (SHBG)** | 0 | 0 |
| **Sortilin** | 0 | 0 |
| **Stem Cell Factor (SCF)** | 0 | 0 |
| **Stromal cell-derived factor-1 (SDF-1)** | 0 | 0 |
| **Superoxide Dismutase 1, soluble (SOD-1)** | 0 | 0 |
| **T Lymphocyte-Secreted Protein I-309 (I-309)** | 73 | 73 |
| **Tamm-Horsfall Urinary Glycoprotein (THP)** | 0 | 0 |
| **T-Cell-Specific Protein RANTES (RANTES)** | 0 | 0 |
| **Tenascin-C (TN-C)** | 0 | 0 |
| **Testosterone, Total** | 1 | 1 |
| **Tetranectin** | 0 | 0 |
| **Thrombin-activable fibrinolysis inhibitor (TAFI)** | 0 | 0 |
| **Thrombospondin-1** | 0 | 0 |
| **Thrombospondin-4 (TSP4)** | 0 | 0 |
| **Thyroglobulin (TG)** | 18 | 18 |
| **Thyroid-Stimulating Hormone (TSH)** | 0 | 0 |
| **Thyroxine-Binding Globulin (TBG)** | 0 | 0 |
| **Tissue Inhibitor of Metalloproteinases 1 (TIMP-1)** | 0 | 0 |
| **Tissue type Plasminogen activator (tPA)** | 3 | 3 |
| **TNF-Related Apoptosis-Inducing Ligand Receptor 3 (TRAIL-R3)** | 0 | 0 |
| **Transforming Growth Factor alpha (TGF-alpha)** | 57 | 57 |
| **Transforming Growth Factor beta-3 (TGF-beta-3)** | 100 | 100 |
| **Transthyretin (TTR)** | 0 | 0 |
| **Trefoil Factor 3 (TFF3)** | 0 | 0 |
| **Tumor Necrosis Factor alpha (TNF-alpha)** | 100 | 100 |
| **Tumor Necrosis Factor beta (TNF-beta)** | 99 | 99 |
| **Tumor necrosis factor receptor 2 (TNFR2)** | 0 | 0 |
| **Tumor Necrosis Factor Receptor I (TNF RI)** | 0 | 0 |
| **Tyrosine kinase with Ig and EGF homology domains 2 (TIE-2)** | 0 | 0 |
| **Urokinase-type Plasminogen Activator (uPA)** | 0 | 0 |
| **Urokinase-type plasminogen activator receptor (uPAR)** | 0 | 0 |
| **Vascular Cell Adhesion Molecule-1 (VCAM-1)** | 0 | 0 |
| **Vascular Endothelial Growth Factor (VEGF)** | 0 | 0 |
| **Vascular Endothelial Growth Factor C (VEGF-C)** | 0 | 0 |
| **Vascular Endothelial Growth Factor Receptor 1 (VEGFR-1)** | 100 | 100 |
| **Vascular Endothelial Growth Factor Receptor 2 (VEGFR-2)** | 0 | 0 |
| **Vascular endothelial growth factor receptor 3 (VEGFR-3)** | 0 | 0 |
| **Vitamin D-Binding Protein (VDBP)** | 0 | 0 |
| **Vitamin K-Dependent Protein S (VKDPS)** | 0 | 0 |
| **Vitronectin** | 0 | 0 |
| **von Willebrand Factor (vWF)** | 0 | 0 |
| **YKL-40** | 0 | 0 |

**Supplementary Table 5. List of all the 187 analytes measured in cohort 8. For each analyte, the percentages of missing values are shown. Analytes are listed in alphabetical order.**

| **Analytes** | **Cohort 8** |
| --- | --- |
| **Adiponectin** | 0 |
| **Adrenocorticotropic Hormone (ACTH)** | 1 |
| **Agouti-Related Protein (AgRP)** | 57 |
| **Alpha 1-Antichymotrypsin** | 1 |
| **Alpha-1 Antitrypsin** | 0 |
| **Alpha-1-Microglobulin** | 0 |
| **Alpha-2 Macroglobulin** | 42 |
| **Alpha-Fetoprotein** | 16 |
| **Amphiregulin** | 74 |
| **Angiopoietin 2 (ANG-2)** | 0 |
| **Angiotensin Converting Enzyme (ACE)** | 0 |
| **Angiotensinogen** | 0 |
| **Apolipoprotein A1** | 0 |
| **Apolipoprotein A2** | 0 |
| **Apolipoprotein A-IV** | 34 |
| **Apolipoprotein B** | 1 |
| **Apolipoprotein CI** | 0 |
| **Apolipoprotein CIII** | 0 |
| **Apolipoprotein D** | 0 |
| **Apolipoprotein E** | 1 |
| **Apolipoprotein H** | 0 |
| **AXL** | 0 |
| **Beta-2 Microglobulin** | 0 |
| **Betacellulin** | 10 |
| **B-Lymphocyte Chemoattractant (BLC)** | 1 |
| **BMP-6** | 73 |
| **Brain-Derived Neurotrophic Factor** | 2 |
| **C Reactive Protein** | 1 |
| **Calbindin** | 66 |
| **Calcitonin** | 79 |
| **Cancer Antigen 125** | 41 |
| **Cancer Antigen 19-9** | 14 |
| **Carcinoembryonic Antigen** | 0 |
| **CD40** | 0 |
| **CD40 Ligand** | 1 |
| **CD5L** | 0 |
| **CgA** | 53 |
| **Ciliary Neurotrophic Factor (CNTF)** | 95 |
| **Clusterin (Apo J)** | 0 |
| **Complement 3** | 0 |
| **Complement Factor H** | 0 |
| **Connective Tissue Growth Factor (CTGF)** | 6 |
| **Cortisol** | 0 |
| **C-peptide** | 5 |
| **Creatine Kinase-MB** | 5 |
| **Cystatin C** | 0 |
| **EGF** | 0 |
| **EGF-R** | 0 |
| **ENA-78** | 0 |
| **Endothelin-1** | 96 |
| **EN-RAGE** | 0 |
| **Eotaxin** | 2 |
| **Eotaxin-3** | 74 |
| **Epiregulin** | 12 |
| **Erythropoietin** | 77 |
| **E-Selectin** | 0 |
| **Factor VII** | 0 |
| **FAS** | 0 |
| **Fas-Ligand** | 0 |
| **Fatty Acid Binding Protein** | 52 |
| **Ferritin** | 0 |
| **Fetuin A** | 0 |
| **FGF basic** | 65 |
| **FGF-4** | 95 |
| **Fibrinogen** | 4 |
| **Follicle Stimulation Hormone (FSH)** | 1 |
| **Gamma-Interferon-induced-Monokine** | 0 |
| **G-CSF** | 1 |
| **Glucagon-like Peptide-1, total (GLP-1 total)** | 96 |
| **Glucagon** | 95 |
| **Glutathione S-Transferase alpha (GST-alpha)** | 1 |
| **GM-CSF** | 57 |
| **GRO-alpha** | 0 |
| **Growth Hormone** | 23 |
| **Haptoglobin** | 0 |
| **HB-EGF** | 0 |
| **HCC-4** | 0 |
| **Heat Shock Protein 60** | 97 |
| **Hepatocyte Growth Factor (HGF)** | 0 |
| **I-309** | 5 |
| **ICAM-1** | 1 |
| **IFN-gamma** | 5 |
| **IgA** | 0 |
| **IgE** | 8 |
| **IGF BP-2** | 0 |
| **IGF-1** | 0 |
| **IgM** | 0 |
| **IL-10** | 59 |
| **IL-11** | 18 |
| **IL-12p40** | 81 |
| **IL-12p70** | 97 |
| **IL-13** | 10 |
| **IL-15** | 24 |
| **IL-16** | 0 |
| **IL-17** | 0 |
| **IL-17E** | 64 |
| **IL-18** | 0 |
| **IL-1alpha** | 73 |
| **IL-1beta** | 75 |
| **IL-1ra** | 1 |
| **IL-2** | 99 |
| **IL-23** | 91 |
| **IL-3** | 12 |
| **IL-4** | 82 |
| **IL-5** | 26 |
| **IL-6** | 51 |
| **IL-6 Receptor** | 0 |
| **IL-7** | 39 |
| **IL-8** | 5 |
| **Insulin** | 8 |
| **Inducible Protein-10 (IP-10)** | 0 |
| **Kidney Injury Molecule-1 (KIM-1)** | 1 |
| **Leptin** | 1 |
| **LH (Luteinizing Hormone)** | 46 |
| **Lipoprotein (a)** | 0 |
| **Lymphotactin** | 99 |
| **MCP-1** | 0 |
| **MCP-2** | 0 |
| **MCP-3** | 86 |
| **MCP-4** | 1 |
| **M-CSF** | 78 |
| **MDC** | 0 |
| **MIF** | 0 |
| **MIP-1alpha** | 0 |
| **MIP-1beta** | 2 |
| **MIP-3 alpha** | 2 |
| **MMP-1** | 0 |
| **MMP10** | 0 |
| **MMP-2** | 31 |
| **MMP-3** | 0 |
| **MMP7** | 0 |
| **MMP-9** | 97 |
| **MMP9 (Total)** | 9 |
| **Myeloid Progenitor Inhibitory Factor 1** | 0 |
| **Myeloperoxidase** | 0 |
| **Myoglobin** | 0 |
| **Neutrophil Gelatinase-Associated Lipocalin (NGAL)** | 2 |
| **NGFb** | 98 |
| **NrCAM** | 0 |
| **Osteopontin** | 0 |
| **PAI-1** | 0 |
| **Pancreatic polypeptide** | 0 |
| **PAPP-A** | 11 |
| **PDGF** | 0 |
| **Progesterone** | 1 |
| **Proinsulin, Intact** | 77 |
| **Proinsulin, Total** | 69 |
| **Prolactin** | 0 |
| **Prostate Specific Antigen, Free** | 26 |
| **Prostatic Acid Phosphatase** | 6 |
| **Protein S** | 0 |
| **Pulmonary and Activation-Regulated Chemokine (PARC)** | 0 |
| **PYY** | 3 |
| **RANTES** | 0 |
| **Resistin** | 0 |
| **S100b** | 85 |
| **Secretin** | 68 |
| **Serum Amyloid P** | 0 |
| **SGOT** | 0 |
| **SHBG** | 0 |
| **SOD** | 0 |
| **Sortilin** | 0 |
| **sRAGE** | 0 |
| **Stem Cell Factor** | 0 |
| **Tamm-Horsfall Protein (THP)** | 0 |
| **Tenascin C** | 0 |
| **Testosterone** | 0 |
| **TGF-alpha** | 27 |
| **TGF-beta 3** | 97 |
| **Thrombopoietin** | 0 |
| **Thrombospondin-1** | 0 |
| **Thymus-Expressed Chemokine (TECK)** | 0 |
| **Thyroid Stimulating Hormone** | 0 |
| **Thyroxine Binding Globulin** | 0 |
| **TIMP-1** | 0 |
| **Tissue Factor** | 95 |
| **TNF RII** | 0 |
| **TNF-alpha** | 53 |
| **TNF-beta** | 78 |
| **TRAIL-R3** | 0 |
| **Transferrin** | 0 |
| **Trefoil Factor 3 (TFF3)** | 0 |
| **TTR (prealbumin)** | 0 |
| **VCAM-1** | 0 |
| **VEGF** | 0 |
| **Vitronectin** | 0 |
| **von Willebrand Factor** | 0 |

**Supplementary Table 6. Overlap of findings with the previous four blood-based protein biomarker studies**

**A)**

| **Markers identified by Schwarz et al 2012^1^**  **(protein names as presented in the paper)** | **Measured in our study?** | **Affected by disease association heterogeneity in our study?** | **Excluded in our analysis?** | **Significant in our study (original 29 analyte panel)?** |
| --- | --- | --- | --- | --- |
| **Betacellulin** | **No** | **na** | **Yes** | **na** |
| **BMP6** | **No** | **na** | **Yes** | **na** |
| **CTGF** | **No** | **na** | **Yes** | **na** |
| **Eotaxin 3** | **No** | **na** | **Yes** | **na** |
| **GM-CSF** | **No** | **na** | **Yes** | **na** |
| **IL17** | **No** | **na** | **Yes** | **na** |
| **LH** | **No** | **na** | **Yes** | **na** |
| **NrCAM** | **No** | **na** | **Yes** | **na** |
| **Alpha 1 Antitrypsin (a1AT)** | **Yes** | **Yes** | **Yes** | **na** |
| **Angiopoietin 2 (ANG2)** | **Yes** | **Yes** | **Yes** | **na** |
| **BDNF** | **Yes** | **Yes** | **Yes** | **na** |
| **Cortisol** | **Yes** | **Yes** | **Yes** | **na** |
| **EGF** | **Yes** | **Yes** | **Yes** | **na** |
| **GST** | **Yes** | **Yes** | **Yes** | **na** |
| **ICAM 1** | **Yes** | **Yes** | **Yes** | **na** |
| **IL5** | **Yes** | **Yes** | **Yes** | **na** |
| **Resistin** | **Yes** | **Yes** | **Yes** | **na** |
| **Sortilin** | **Yes** | **Yes** | **Yes** | **na** |
| **Thrombopoietin (TPO)** | **Yes** | **Yes** | **Yes** | **na** |
| **Alpha 2 Macroglobulin (A2M)** | **Yes** | **No** | **No** | **Yes** |
| **CEA** | **Yes** | **No** | **No** | **Yes** |
| **Factor VII** | **Yes** | **No** | **No** | **Yes** |
| **FSH** | **Yes** | **No** | **No** | **Yes** |
| **Haptoglobin (HPT)** | **Yes** | **No** | **No** | **Yes** |
| **IGFBP 2** | **Yes** | **No** | **No** | **Yes** |
| **IL10** | **Yes** | **No** | **No** | **Yes** |
| **MIF** | **Yes** | **No** | **No** | **Yes** |
| **PP** | **Yes** | **No** | **No** | **Yes** |
| **SCF** | **Yes** | **No** | **No** | **Yes** |
| **SGOT** | **Yes** | **No** | **No** | **Yes** |
| **CD40L** | **Yes** | **No** | **No** | **No** |
| **PAP** | **Yes** | **No** | **No** | **No** |
| **RANTES (C-C motif chemokine 5)** | **Yes** | **No** | **No** | **No** |
| **Thrombospondin 1 (TSP1)** | **Yes** | **No** | **No** | **No** |

B)

| **Markers identified by Schwarz et al 2013^2^**  **(protein names as presented in the paper)** | **Measured in our study?** | **Affected by disease association heterogeneity in our study?** | **Excluded in our analysis?** | **Significant in our study (original 29 analyte panel)?** |
| --- | --- | --- | --- | --- |
| **Amphiregulin** | **No** | **na** | **Yes** | **na** |
| **Betacellulin** | **No** | **na** | **Yes** | **na** |
| **Bone morphogenetic protein (BMP)-6** | **No** | **na** | **Yes** | **na** |
| **Connective tissue growth factor (CTGF)** | **No** | **na** | **Yes** | **na** |
| **Endothelin-1** | **No** | **na** | **Yes** | **na** |
| **Erythropoietin** | **No** | **na** | **Yes** | **na** |
| **Fas ligand** | **No** | **na** | **Yes** | **na** |
| **FGF basic** | **No** | **na** | **Yes** | **na** |
| **Fibroblast growth factor (FGF)-4** | **No** | **na** | **Yes** | **na** |
| **IL-1 alpha** | **No** | **na** | **Yes** | **na** |
| **IL-1 beta** | **No** | **na** | **Yes** | **na** |
| **IL-11** | **No** | **na** | **Yes** | **na** |
| **IL-12p40** | **No** | **na** | **Yes** | **na** |
| **IL-12p70** | **No** | **na** | **Yes** | **na** |
| **IL-15** | **No** | **na** | **Yes** | **na** |
| **IL-2** | **No** | **na** | **Yes** | **na** |
| **IL-4** | **No** | **na** | **Yes** | **na** |
| **Interferon (IFN)-gamma** | **No** | **na** | **Yes** | **na** |
| **Luteinizing hormone (LH)** | **No** | **na** | **Yes** | **na** |
| **TGF-beta3** | **No** | **na** | **Yes** | **na** |
| **TNF-beta** | **No** | **na** | **Yes** | **na** |
| **Transforming growth factor (TGF)-alpha** | **No** | **na** | **Yes** | **na** |
| **Tumor necrosis factor (TNF)-alpha** | **No** | **na** | **Yes** | **na** |
| **Growth hormone** | **No** | **na** | **Yes** | **na** |
| **Angiopoietin 2** | **Yes** | **Yes** | **Yes** | **na** |
| **Angiotensinogen** | **Yes** | **Yes** | **Yes** | **na** |
| **Brain-derived neurotrophicfactor (BDNF)** | **Yes** | **Yes** | **Yes** | **na** |
| **Cortisol** | **Yes** | **Yes** | **Yes** | **na** |
| **Epidermal growth factor (EGF)** | **Yes** | **Yes** | **Yes** | **na** |
| **IL-16** | **Yes** | **Yes** | **Yes** | **na** |
| **IL-5** | **Yes** | **Yes** | **Yes** | **na** |
| **Insulin** | **Yes** | **Yes** | **Yes** | **na** |
| **Progesterone** | **Yes** | **Yes** | **Yes** | **na** |
| **Prolactin** | **Yes** | **Yes** | **Yes** | **na** |
| **Resistin** | **Yes** | **Yes** | **Yes** | **na** |
| **Thrombopoietin** | **Yes** | **Yes** | **Yes** | **na** |
| **Vascular endothelial growth factor (VEGF)** | **Yes** | **Yes** | **Yes** | **na** |
| **Platelet-derived growth factor (PDGF)** | **Yes** | **No** | **No** | **No** |
| **IL-7** | **Yes** | **No** | **No** | **No** |
| **IL-18** | **Yes** | **No** | **No** | **No** |
| **HB-epidermal growth factor** | **Yes** | **No** | **No** | **No** |
| **CD40 ligand** | **Yes** | **No** | **No** | **No** |
| **Adiponectin** | **Yes** | **No** | **No** | **No** |
| **Thyroid stimulating hormone (TSH)** | **Yes** | **No** | **No** | **Yes** |
| **Testosterone** | **Yes** | **No** | **No** | **Yes** |
| **Pancreatic polypeptide** | **Yes** | **No** | **No** | **Yes** |
| **Macrophage migration inhibitory factor (MIF)** | **Yes** | **No** | **No** | **Yes** |
| **Leptin** | **Yes** | **No** | **No** | **Yes** |
| **Interleukin (IL)-10** | **Yes** | **No** | **No** | **Yes** |
| **IL-8** | **Yes** | **No** | **No** | **Yes** |
| **IL-1ra** | **Yes** | **No** | **No** | **Yes** |
| **IL-13** | **Yes** | **No** | **No** | **Yes** |
| **Follicle stimulating hormone (FSH)** | **Yes** | **No** | **No** | **Yes** |

**C**)

| **Markers identified by Domenici et al^3^ by univariate analysis**  **(protein names as presented in the paper)** | **Measured in our study?** | **Affected by disease association heterogeneity in our study?** | **Excluded in our analysis?** | **Significant in our study (original 29 analyte panel)?** |
| --- | --- | --- | --- | --- |
| **Cancer Antigen 125** | **No** | **na** | **Yes** | **na** |
| **Complement 3** | **No** | **na** | **Yes** | **na** |
| **Endothelin-1** | **No** | **na** | **Yes** | **na** |
| **Fibrinogen** | **No** | **na** | **Yes** | **na** |
| **GM-CSF** | **No** | **na** | **Yes** | **na** |
| **Growth Hormone** | **No** | **na** | **Yes** | **na** |
| **IgE** | **No** | **na** | **Yes** | **na** |
| **IL-12p40** | **No** | **na** | **Yes** | **na** |
| **IL-12p70** | **No** | **na** | **Yes** | **na** |
| **IL-15** | **No** | **na** | **Yes** | **na** |
| **IL-1alpha** | **No** | **na** | **Yes** | **na** |
| **IL-4** | **No** | **na** | **Yes** | **na** |
| **MMP-2** | **No** | **na** | **Yes** | **na** |
| **MMP-9** | **No** | **na** | **Yes** | **na** |
| **Tissue Factor** | **No** | **na** | **Yes** | **na** |
| **TNF-beta** | **No** | **na** | **Yes** | **na** |
| **Alpha-1 Antitrypsin** | **Yes** | **Yes** | **Yes** | **na** |
| **Apolipoprotein CIII** | **Yes** | **Yes** | **Yes** | **na** |
| **Brain-Derived Neurotrophic Factor** | **Yes** | **Yes** | **Yes** | **na** |
| **C Reactive Protein** | **Yes** | **Yes** | **Yes** | **na** |
| **EGF** | **Yes** | **Yes** | **Yes** | **na** |
| **Glutathione S-Transferase** | **Yes** | **Yes** | **Yes** | **na** |
| **ICAM-1** | **Yes** | **Yes** | **Yes** | **na** |
| **IL-16** | **Yes** | **Yes** | **Yes** | **na** |
| **Insulin** | **Yes** | **Yes** | **Yes** | **na** |
| **Thrombopoietin** | **Yes** | **Yes** | **Yes** | **na** |
| **Thyroxine Binding Globulin** | **Yes** | **Yes** | **Yes** | **na** |
| **TIMP-1** | **Yes** | **Yes** | **Yes** | **na** |
| **TNF RII** | **Yes** | **Yes** | **Yes** | **na** |
| **VEGF** | **Yes** | **Yes** | **Yes** | **na** |
| **Alpha-2 Macroglobulin** | **Yes** | **No** | **No** | **Yes** |
| **Apolipoprotein A1** | **Yes** | **No** | **No** | **Yes** |
| **Apolipoprotein H** | **Yes** | **No** | **No** | **Yes** |
| **Carcinoembryonic Antigen** | **Yes** | **No** | **No** | **Yes** |
| **Eotaxin** | **Yes** | **No** | **No** | **Yes** |
| **Factor VII** | **Yes** | **No** | **No** | **Yes** |
| **IgA** | **Yes** | **No** | **No** | **Yes** |
| **IL-10** | **Yes** | **No** | **No** | **Yes** |
| **IL-13** | **Yes** | **No** | **No** | **Yes** |
| **IL-8** | **Yes** | **No** | **No** | **Yes** |
| **Leptin** | **Yes** | **No** | **No** | **Yes** |
| **Stem Cell Factor** | **Yes** | **No** | **No** | **Yes** |
| **VCAM-1** | **Yes** | **No** | **No** | **Yes** |
| **von Willebrand Factor** | **Yes** | **No** | **No** | **Yes** |
| **Adiponectin** | **Yes** | **No** | **No** | **No** |
| **ENA-78** | **Yes** | **No** | **No** | **No** |
| **IL-18** | **Yes** | **No** | **No** | **No** |
| **IL-3** | **Yes** | **No** | **No** | **No** |
| **MCP-1** | **Yes** | **No** | **No** | **No** |
| **MDC** | **Yes** | **No** | **No** | **No** |
| **MIP-1beta** | **Yes** | **No** | **No** | **No** |
| **PAI-1** | **Yes** | **No** | **No** | **No** |
| **Prostatic Acid Phosphatase** | **Yes** | **No** | **No** | **No** |
| **RANTES** | **Yes** | **No** | **No** | **No** |
| **Serum Amyloid P** | **Yes** | **No** | **No** | **No** |

**D**)

| **Markers identified by Perkins et al 2014^4^** | **Measured in our study?** | **Affected by disease association heterogeneity in our study?** | **Excluded in our analysis?** | **Significant in our study (original 29 analyte panel)?** |
| --- | --- | --- | --- | --- |
| **Interleukin-1 beta (IL-1 beta)** | **No** | **na** | **Yes** | **na** |
| **Immunoglobulin E (IgE)** | **No** | **na** | **Yes** | **na** |
| **Growth Hormone (GH)** | **No** | **na** | **Yes** | **na** |
| **Malondialdehyde-Modified**  **Low-Density Lipoprotein (MDA-LDL)** | **NI** | **na** | **Yes** | **na** |
| **Matrix Metalloproteinase-7 (MMP-7)** | **NI** | **na** | **Yes** | **na** |
| **Uromodulin** | **NI** | **na** | **Yes** | **na** |
| **Apolipoprotein D (Apo D)** | **NI** | **na** | **Yes** | **na** |
| **KIT ligand** | **NI** | **na** | **Yes** | **na** |
| **Chemokine ligand 8** | **NI** | **na** | **Yes** | **na** |
| **Cortisol** | **Yes** | **Yes** | **Yes** | **na** |
| **Resistin** | **Yes** | **Yes** | **Yes** | **na** |
| **Interleukin-7 (IL-7)** | **Yes** | **No** | **No** | **No** |
| **Thyroid-Stimulating Hormone (TSH)** | **Yes** | **No** | **No** | **Yes** |
| **Factor VII** | **Yes** | **No** | **No** | **Yes** |
| **Interleukin-8 (IL-8)** | **Yes** | **No** | **No** | **Yes** |

**NI:** analyte not measured in the Myriad-RBM assay version used in this study; **na:** not applicable; **Measured in our study** refers to whether the analyte survived QC testing. If >30% missing value then the analyte is excluded from our subsequent analyses and is classified as not measured in our study.

**References:**

1. Schwarz E, Guest PC, Rahmoune H, Harris LW, Wang L, Leweke FM *et al.* Identification of a biological signature for schizophrenia in serum. *Mol Psychiatry* 2012; **17**(5)**:** 494-502.

2. Schwarz E, van Beveren NJ, Ramsey J, Leweke FM, Rothermundt M, Bogerts B *et al.* Identification of Subgroups of Schizophrenia Patients With Changes in Either Immune or Growth Factor and Hormonal Pathways. *Schizophr Bull* 2013.

3. Domenici E, Wille DR, Tozzi F, Prokopenko I, Miller S, McKeown A *et al.* Plasma protein biomarkers for depression and schizophrenia by multi analyte profiling of case-control collections. *PLoS One* 2010; **5**(2)**:** e9166.

4. Perkins DO, Jeffries CD, Addington J, Bearden CE, Cadenhead KS, Cannon TD *et al.* Towards a Psychosis Risk Blood Diagnostic for Persons Experiencing High-Risk Symptoms: Preliminary Results From the NAPLS Project. *Schizophr Bull* 2014.
